# Neuronal K^+^-Cl^-^ cotransporter KCC2 as a promising drug target for epilepsy treatment

**DOI:** 10.1038/s41401-023-01149-9

**Published:** 2023-09-13

**Authors:** Erin McMoneagle, Jin Zhou, Shiyao Zhang, Weixue Huang, Sunday Solomon Josiah, Ke Ding, Yun Wang, Jinwei Zhang

**Affiliations:** 1 Institute of Biomedical and Clinical Sciences, Medical School, Faculty of Health and Life Sciences, University of Exeter, Hatherly Laboratories, Streatham Campus, Exeter, EX4 4PS UK; 2 Department of Neurology, Institutes of Brain Science, State Key Laboratory of Medical Neurobiology and MOE Frontiers Center for Brain Science, Institute of Biological Science, Zhongshan Hospital, Fudan University, Shanghai, 200032 China; 3 Institute of Cardiovascular Diseases, Xiamen Cardiovascular Hospital Xiamen University, School of Medicine, Xiamen University, Xiang’an Nan Lu, Xiamen, 361102 China; 4 State Key Laboratory of Chemical Biology, Research Center of Chemical Kinomics, Shanghai Institute of Organic Chemistry, Chinese Academy of Sciences, Shanghai, 200032 China

**Keywords:** epilepsy, GABAergic inhibition, K^+^-Cl^-^ cotransporter KCC2, chloride homeostasis, signaling regulatory pathways, small molecular compounds

## Abstract

Epilepsy is a prevalent neurological disorder characterized by unprovoked seizures. γ-Aminobutyric acid (GABA) serves as the primary fast inhibitory neurotransmitter in the brain, and GABA binding to the GABA_A_ receptor (GABA_A_R) regulates Cl^-^ and bicarbonate (HCO_3_^-^) influx or efflux through the channel pore, leading to GABAergic inhibition or excitation, respectively. The neuron-specific K^+^-Cl^-^ cotransporter 2 (KCC2) is essential for maintaining a low intracellular Cl^-^ concentration, ensuring GABA_A_R-mediated inhibition. Impaired KCC2 function results in GABAergic excitation associated with epileptic activity. Loss-of-function mutations and altered expression of KCC2 lead to elevated [Cl^-^]_i_ and compromised synaptic inhibition, contributing to epilepsy pathogenesis in human patients. KCC2 antagonism studies demonstrate the necessity of limiting neuronal hyperexcitability within the brain, as reduced KCC2 functioning leads to seizure activity. Strategies focusing on direct (enhancing KCC2 activation) and indirect KCC2 modulation (altering KCC2 phosphorylation and transcription) have proven effective in attenuating seizure severity and exhibiting anti-convulsant properties. These findings highlight KCC2 as a promising therapeutic target for treating epilepsy. Recent advances in understanding KCC2 regulatory mechanisms, particularly via signaling pathways such as WNK, PKC, BDNF, and its receptor TrkB, have led to the discovery of novel small molecules that modulate KCC2. Inhibiting WNK kinase or utilizing newly discovered KCC2 agonists has demonstrated KCC2 activation and seizure attenuation in animal models. This review discusses the role of KCC2 in epilepsy and evaluates its potential as a drug target for epilepsy treatment by exploring various strategies to regulate KCC2 activity.

## KCC2 and Chloride Homeostasis

### The cell-type and regional expression of KCC2 and NKCC1

The K^+^-Cl^-^ cotransporter KCC2 and the Na^+^-K^+^-Cl^-^ cotransporter 1 (NKCC1) belong to the cation chloride cotransporters (CCCs) family, and they are encoded by *Slc12a5* and *Slc12a2*, respectively. NKCC1 is expressed ubiquitously in various cell types, including central and peripheral neurons, as well as glial cells [1, 2]. In contrast, KCC2 is selectively expressed on the plasma membrane of somata and dendrites of pyramidal neurons and interneurons in the hippocampus and neocortex [3, 4]. Additionally, KCC2 expression has been observed in the adult animal retina [5, 6], as well as in INS-1E β-cell lines or glucagon-positive α cells of pancreatic islets, where it regulates insulin secretion [7, 8]. However, KCC2 exhibits a more nerve-specific expression pattern compared to NKCC1. Therefore, it is more reasonable to consider KCC2 as a potential drug target for the treatment of brain disorders.

### Chloride homeostasis and the role of normal KCC2 functioning

Chloride (Cl^-^) homeostasis plays a crucial role in determining the polarity of signaling within the central nervous system (CNS). The primary fast inhibitory neurotransmitter, γ-aminobutyric acid (GABA), binds to the ionotropic GABA_A_ receptor (GABA_A_R) located on the postsynaptic neuronal membrane [9]. Upon GABA binding to its receptor, the activation of GABA_A_R leads to the influx or efflux of Cl^-^ and bicarbonate (HCO_3_^-^) through the channel pore, resulting in GABAergic inhibition or excitation, respectively [9–11]. This GABAergic signaling is dependent upon the intracellular Cl^-^ concentration, which determines the reversal potential for the GABA_A_R (E_GABA_) [10, 11]. When the intracellular Cl^-^ concentration is high, E_GABA_ is more depolarized relative to the resting membrane potential, resulting in neuronal depolarization on GABA_A_ activation [11]. Conversely, when intracellular Cl^-^ is low, E_GABA_ is more hyperpolarized relative to the resting membrane potential, leading to GABA-mediated hyperpolarization and GABAergic inhibition [11].

**Fig. 1:**
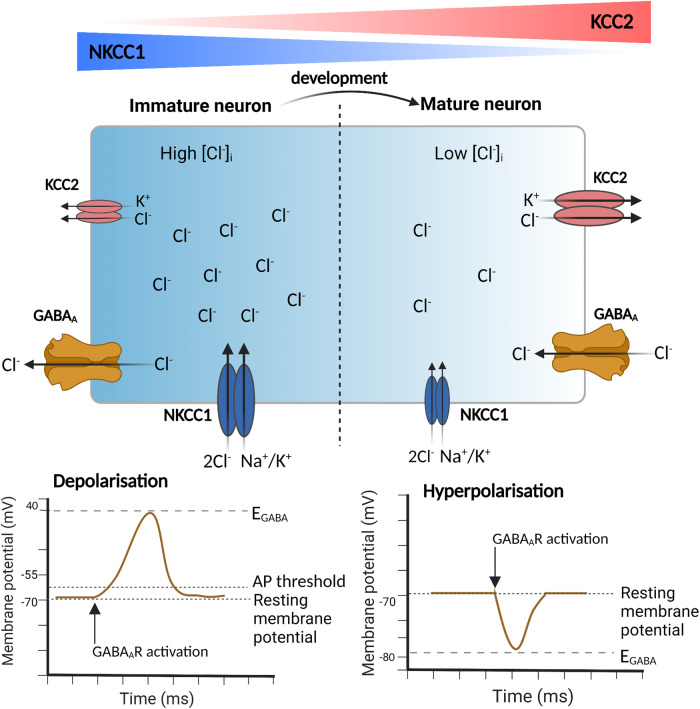
Developmental shifts in KCC2 and NKCC1 expression levels modulate GABAergic signaling from depolarizing to hyperpolarizing. NKCC1 imports Cl^-^ into neurons, while KCC2 exports Cl^-^. High NKCC1 expression in immature neurons leads to elevated intracellular Cl^-^ levels, resulting in a depolarized E_GABA_ relative to the resting membrane potential. This triggers Cl^-^ efflux through GABA_A_Rs, causing membrane depolarization. However, as neurons mature, KCC2 expression increases, and NKCC1 expression decreases. Increased KCC2 activity lowers intracellular Cl^-^ levels, establishing a hyperpolarized E_GABA_ compared to the resting membrane potential. This induces inward GABAergic Cl^-^ currents, hyperpolarizing mature neurons. Diagram created using BioRender.com. [Cl^-^]_i_ intracellular chloride concentration, NKCC1 Na^+^-K^+^-Cl^-^ cotransporter 1, KCC2 K^+^-Cl^-^ cotransporter 2, [Cl^-^]_i_ Intracellular chloride concentration, GABA_A_R γ-aminobutyric acid receptor type A, Na^+^ sodium, K^+^ Potassium; and AP action potential.

Neuronal Cl^-^ homeostasis is regulated by KCC2 and NKCC1. NKCC1 drives Cl^-^ into the neuron using the Na^+^ gradient, while KCC2 extrudes Cl^-^, driven by the K^+^ gradient generated by the active transporter Na^+^/K^+^/ATPase [11]. However, during neuronal development, NKCC1 and KCC2 are reciprocally expressed and undergo changes (Fig. 1) [12, 13]. In early neuronal development, NKCC1 activity predominates with high expression levels [13]. As neuronal maturation progresses, KCC2 expression increases, becoming the prominent Cl^-^ extruder in mature neurons [13–15]. This shift in KCC2 and NKCC1 expression corresponds to a developmental transition from GABA-mediated depolarization to hyperpolarization [11, 13, 14, 16]. Therefore, maintaining a balance between the activities of KCC2 and NKCC1 is crucial for GABAergic inhibition.

### Epilepsy and the involvement of disrupted Cl^-^ homeostasis

Epilepsy is a chronic neurological disorder characterized by the occurrence of two or more recurrent seizures unprovoked by systemic or acute neurological insult [17]. Seizures occur due to a hyperexcitable neuronal network and synchronization [18]. Epileptogenesis is the process by which a ‘normal’ neuronal network switches to a hyperexcitable network. This hyperexcitable state within the brain can result from either increased excitatory neurotransmission or decreased inhibitory neurotransmission [18].

Epilepsy is believed to result from an imbalance in the electrical activity of the brain. Disrupted Cl^-^ homeostasis is one of the factors that can contribute to the development and occurrence of seizures in epilepsy [19]. Chloride ions play a crucial role in maintaining the balance of electrical signals in neurons. Under normal circumstances, chloride ions are primarily maintained at low levels inside neurons, creating a negative membrane potential. This negative potential helps to stabilize the resting state of neurons and prevents excessive excitation. In epilepsy, disrupted Cl^-^ homeostasis occurs due to impaired regulation of chloride ions [19]. This can result from altered expression or functionality of transporters of KCC2 or NKCC1, which respectively promote the exit and entry of chloride ions in neurons [3]. Such changes in transporter levels or functionality contribute to the chloride ion imbalance seen in epilepsy.

Furthermore, in some forms of epilepsy, there can be alterations in the GABAergic system, including changes in GABA receptors or GABA release, which can disrupt the normal inhibitory function of GABA and lead to increased neuronal excitability [20]. The C-terminus of KCC2 contains a region called the isotonic (ISO) domain, which is necessary for KCC2 to facilitate GABAergic hyperpolarizing signaling [21]. Due to its role as a key modulator in inhibitory GABAergic signaling, KCC2 has been implicated in various neuropathological conditions involving inhibitory dysfunction, such as Huntington’s disease, Rett syndrome, spinal cord injury, autism, and epilepsy [19, 22–26].

This review focuses on the reduced inhibitory GABAergic signaling caused by reduced KCC2-dependent chloride extrusion, leading to a high intracellular Cl^-^ concentration in neurons. This loss of GABAergic inhibition underlies epileptogenesis and the development of seizures [27]. Epileptic seizures are classified into three main groups: focal (seizure activity is localized in one region of the brain in one hemisphere), generalized (seizure activity occurs over both hemispheres), and unknown [28]. The diagnosis of epilepsy is based on the presence of spike-wave activity on an electroencephalogram (EEG) as well as the clinical presentation of symptoms [29].

### The importance of identifying a new therapeutic target for epileptic treatment

Approximately 50 million people live with epilepsy, making it one of the most common neurological diseases. The World Health Organization (WHO) recognizes epilepsy as a major public health concern. Currently, the main therapeutic interventions used to prevent seizure generation are anti-epileptic drugs (AEDs). However, despite the effectiveness of currently available AEDs for many epileptic patients, approximately one-third of patients remain drug-resistant [30]. These individuals are considered to have refractory epilepsy [30]. The majority of anti-epileptic drugs act by enhancing GABAergic inhibition via GABA_A_Rs [31]. For instance, two commonly used AEDs, benzodiazepines, and phenobarbital, primarily potentiate GABA_A_R activity to increase neuronal inhibition in the brain, particularly in the treatment of status epilepticus (severe epileptic activity that is considered a medical emergency) [32–34].

Given that current treatment options are ineffective for one-third of epileptic patients, there is a clinical need for the development of novel therapeutic targets [30]. Recently, a loop diuretic, bumetanide, which inhibits NKCC1 activation by binding to it, has been proposed as a promising therapeutic agent and has undergone clinical trials for epileptic treatment [35, 36]. Although bumetanide has shown some success as an epileptic treatment, it has been suggested that KCC2 may be more desirable than NKCC1 to reduce neuronal intracellular Cl^-^ concentration. This is mainly because NKCC1 is widely expressed in the periphery (unlike KCC2), such as in the kidney, leading to side effects like hypokalaemia [37–40]. Therefore, direct targeting of KCC2 with agonists would potentially result in fewer side effects due to greater target specificity. Current research focuses on the role of KCC2 in epileptic pathology and identifying potential mechanisms to augment KCC2 activity for reducing neuronal excitability. However, it remains unclear whether KCC2 would be a clinically viable therapeutic drug target for epileptic treatment.

The aim of this review is to provide insights into the role of KCC2 in epileptogenesis and evaluate the potential of targeting KCC2 with various therapeutic agents for the treatment of epilepsy. This will be achieved by examining the current evidence regarding the involvement of reduced KCC2 function in epileptic activity, KCC2 dysfunction in epileptic patients, and discussing the existing strategies for direct and indirect modulation of KCC2. By addressing these areas, this review seeks to answer the research question: Is there sufficient evidence to support KCC2 as a promising drug target for epilepsy treatment?

## The role of KCC2 in epileptogenesis

**Fig. 2:**
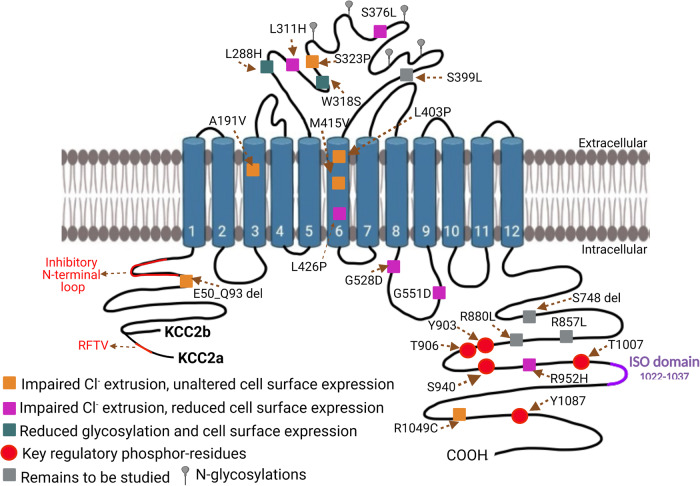
Schematic representation of *SLC12A5* mutations associated with human epilepsy and key regulatory phosphorylation sites. KCC2 consists of two N-terminal splice isoforms, KCC2a and KCC2b, which comprise 12 transmembrane (TM) domains, 11 loops, N-terminus, and C-terminus. KCC2a contains an additional 23-amino-acid sequence with a conserved SPAK/OSR1-binding domain (RFTV). The N-terminal domains of KCC2a (V81-N107) and KCC2b (A66-N83) exhibit an autoinhibitory function, preventing intracellular solvent access to the ion-binding sites within TM1, 3, 6, and 8. The purple region represents the ISO domain, essential for hyperpolarizing GABAergic signaling. Within the intracellular carboxy-terminal domain (CTD), crucial regulatory phosphorylation sites of KCC2, including WNK-SPAK/OSR1 kinase sites (Threonine T906, T1007), PKC phosphorylation sites (Serine S940), and Src family kinase phosphorylation sites (Tyrosine Y903, Y1087), are located. The figure key outlines the mutation phenotypes and implications of the phosphorylation sites. The diagram was created using BioRender.com. SLC12 solute carrier family 12, KCC2 K^+^-Cl^-^ cotransporter 2, TM transmembrane, GABA γ-aminobutyric acid, CTD carboxy-terminal domain, WNK With-No-Lysine (K) kinases, SPAK SPS/Ste20-related proline-alanine-rich kinase, OSR1 oxidative stress-responsive kinase 1, PKC protein kinase C, and ISO isotonic.

Preclinical studies investigating KCC2 antagonism have demonstrated that impaired KCC2 function leads to neuronal hyperexcitability and epileptic-like activity. This suggests that reduced KCC2 activity may be involved in the mechanisms underlying increased excitability in the brains of epileptic patients. The identification of various KCC2 mutations (encoded by *SLC12A5*) has further highlighted the association between KCC2 dysfunction and the development of epilepsy [41–46]. A summary of the KCC2 mutations discovered in human epilepsy can be found in Table 1 [47], with their localization shown in Fig. 2. In addition to preclinical studies, analyzing KCC2 dysfunction in humans is necessary to determine whether targeting KCC2 would be an effective therapeutic strategy in the treatment of epilepsy.

**Table 1:** Current KCC2 (*SLC12A5*) mutations identified in human epileptic patients, and their localisation/expression/trafficking/activity changes.

NT^a^ change	AA^b^ change	Type	Inheritance	Pheno-type	Localisation	Functional validation	Reference
c.2855 G > A	p.R952H	Missense	AD^c^	IGE^d^; Febrile seizures	Cytoplasmic C-terminus	Impaired Cl^-^ extrusion, reduced cell surface expression, impaired ability to form dendritic spines.	[44, 45]
c.3145 C > T	p.R1049C	Missense	AD	IGE	Cytoplasmic C-terminus	Reduced glycosylation and cell surface expression	[44]
c.1277 T > C	p.L426P	Missense	AR^f^, CH^g^	EIMFS^h^	Within TM6^e^	Impaired Cl^-^ extrusion, reduced glycosylation and cell surface expression.	[43]
c.1583 G > A	p.G528D	Missense	AR, CH	EIMFS	Intracellular loop between TM8-TM9	Impaired Cl^-^ extrusion	[43]
c.1625G>A	p.G551D	Missense	AR, CH	EIMFS	Intracellular loop between TM8-TM9	Impaired Cl^-^ extrusion, reduced glycosylation and cell surface expression.	[43]
c.932 T > A	p.L311H	Missense	AR, homozygous	EIMFS	Large extracellular loop between TM5 & TM6	Impaired Cl^-^ extrusion, reduced cell surface expression	[43]
c.279+1 G > C	p.E50_Q93del	Deletion	AR, CH	EIMFS	Lacks 44 amino acids including the N-terminal inhibitory loop	Impaired Cl^-^ extrusion, unaltered cell surface expression	[42]
c.572 C > T	p.A191V	Missense	AR, CH	EIMFS	Within TM3	Impaired Cl^-^ extrusion, unaltered cell surface expression	[42]
c.1208 T > C	p.L403P	Missense	AR, CH	EIMFS	Within TM6	Impaired Cl^-^ extrusion	[42, 43]
c.1243 A > G	p.M415V	Missense	AR, CH	EIMFS	Within TM6	Impaired Cl^-^ extrusion, unaltered cell surface expression	[42]
c.967 T > C	p.S323P	Missense	AR, CH	EIMFS	Large extracellular loop between TM5 & TM6	Impaired Cl^-^ extrusion, unaltered cell surface expression	[42]
c.863 T > A	p.L288H	Missense	AR, CH	EIMFS	Large extracellular loop between TM5 & TM6	Reduced glycosylation and cell surface expression	[42]
c.953 G > C	p.W318S	Deletion	AR, CH	EIMFS	Large extracellular loop between TM5 & TM6	Reduced glycosylation and cell surface expression	[42]
c.2242_2244del	p.S748del	Missense	AR, CH	EIMFS	Cytoplasmic C-terminus	Remains to be studied	[42]
c.2570 G > T	p.R857L	Missense	AR, CH	EIMFS	Cytoplasmic C-terminus	Remains to be studied	[42]
c.1196 C.T	p.S399L	Missense	AR, CH	EIMFS	Exact location unverified	Remains to be studied	[49]
c.2639 G > T	p.R880L	Missense	AR, CH	EIMFS	Cytoplasmic C-terminus	Remains to be studied	[49]
c.1417 G > A	p.V473I	Missense	AD	IGE	Exact location unverified	Remains to be studied	[41, 46]
C > G / C > T		rs2297201 polymorphism	CH, homozygous	FS^i^		Remains to be studied	[70]

^a^NT, Nucleotide change.

^b^AA, Amino acid.

^c^AD, Autosomal dominant.

^d^IGE, Idiopathic generalised epilepsy.

^e^TM, Transmembrane domain.

^f^AR, Autosomal recessive.

^g^CH, Compound heterozygous.

^h^EIMFS, Epilepsy of infancy with migrating focal seizures.

^i^FS, Febrile seizures.

### Epilepsy of infancy with migrating focal seizures

Evidence from epileptic patients indicates that various KCC2 mutations result in impaired chloride extrusion. This impaired Cl^-^ extrusion has been proposed to underlie the mechanism by which *SLC12A5* mutations cause epilepsy of infancy with migrating focal seizures (EIMFS) [42, 43]. Saitsu et al. studied patients from families with EIMFS who had been diagnosed with severe infantile epilepsy syndrome [42]. Through whole-exome sequencing of ten sporadic cases and one familial case of EIMFS, Saitsu et al. discovered heterozygous *SLC12A5* mutations in two families [42]. These mutations include c.279 + 1 G > C, which causes skipping of exon 3 in the mRNA transcript (p.E50_Q93del), c.572 C > T (p.A191V) in two separate individuals, and both c.967 T > C (p.S323P) and c.1243 A > G (p.M415V) in another individual [42]. Targeted resequencing of 141 patients with infantile epilepsy from 526 epileptic patients identified a further two heterozygous mutations of c.953 G > C (p.W318S) and c.2242_2244del (p.S748del) in another individual [42]. Functional analysis showed that some of the above mutations (E50_Q93del, A191V, S323P, M415V) suppressed Cl^-^ extrusion to varying degrees, without affecting KCC2 cell surface expression [42]. Cells exhibiting the pair of KCC2 mutations S323P and M415V demonstrated a significantly greater positive shift of ECl (17.6 mV) compared to cells expressing E50_Q93del and A191V mutations (12 mV) (WT: −59.9  ±  2.9 mV; S323P and M415V: −42.3  ±  3.9 mV, *P* < 0.01; E50_Q93del and A191V: −47.9 ±  3.1 mV) [42]. This positive shift may correspond to an increase in excitatory GABA signaling [19]. However, the assessment of the change in ECl by individual mutations suggested that E50_Q93del and M415V were the most significant mutations underlying the increase in intracellular Cl^-^ concentration, as the other mutations did not reach a significant change in ECl compared to wild type (WT). A potential reason for this difference could be the different localizations of these mutations (Fig. 2). However, the authors acknowledged that the immunoblotting assay used would not have been able to detect minor changes in KCC2 surface expression level due to low sensitivity [42]. This may explain the subtle differences in the severity of Cl^-^ impairment between the mutations and WT KCC2 [42]. Analyzing KCC2 surface expression using cold-adapted trypsin could have been utilized in addition to surface biotinylation to check the KCC2 surface expression [48]. Despite this, a follow-up study also found attenuated neuronal Cl^-^ extrusion without altered KCC2 surface expression in EIMFS patients with *SLC12A5* mutations [49]. This suggests that another explanation, such as impairment to KCC2’s intrinsic transport properties, could underlie the impaired Cl^-^ extrusion mechanisms of KCC2.

EIMFS patients with *SLC12A5* mutations have also shown altered KCC2 surface expression. Stödberg et al. discovered biallelic *SLC12A5* loss-of-function mutations through screening 42 patients with EIMFS [43]. Functional analysis of all three mutations identified by Stödberg et al. demonstrated diminished Cl^-^ extrusion as well as reduced cell surface expression [43] (Table 2). This differs somewhat from the results indicated by Saitsu et al. and Saito et al. [42, 43, 49]. However, it has been proposed that Stödberg et al.’s assessment of Cl^-^ extrusion may be inaccurate due to using a whole-cell pipette solution containing 110 mM Cs^+^, instead of K^+^, to measure E_Cl_. [42, 43]. This would result in KCC2 being unable to extrude Cl^-^ as KCC2 requires a K^+^ gradient for Cl^-^ extrusion [42]. The use of gramicidin perforated patch clamp electrophysiology, as used by Saitsu et al., would have been a more accurate method, providing a high-precision estimation of intracellular Cl^-^ concentration at a single-cell level in the presence of K^+^ [42, 50]. Despite this, the difference in KCC2 surface expression between the different mutations may be due to the differences in their genic location. For example, the mutations examined by Saitsu et al. were located within the N-terminal domain (p.E50_Q93del), transmembrane domains (p.A191V and p.M415V), and the large extracellular loop (p.S323P) [42]. In comparison, Stödberg et al. discovered mutations located in the transmembrane domain (p.2426 P) and intracellular loops (p.L331H and p.G551D) [42, 43]. Mutations discovered by Stödberg et al. may have a greater effect on KCC2 trafficking to the cell surface [43]. The literature, therefore, highlights the need for further studies to assess whether these mutations affect either or both the intrinsic properties and trafficking of KCC2.

**Table 2:** Small molecular compounds or a CRISPRa system used in modulating KCC2 activity via its direct and indirect modulation on its function or epileptic seizure activity.

Molecule	Chemical structure	Target	Model	Findings	Reference
KCC2 inhibitors or antagonists					
VU0463271		KCC2	C57BL/6 mice subicular slices or hippocampal slices (ex vivo)	Generated hypersynchronous discharged and induced status epilepticus.	[167]; [150]
Furosemide		KCC2	Sprague–Dawley (SD) rats (in vivo)	Prevented membrane KCC2 downregulation during acute seizure induction, restored KCC2-mediated GABA inhibition, and interrupted the progression from acute seizure to epileptogenesis.	[168]
KCC2 activators or agonists					
OV350	N/A	KCC2	Cultured forebrain neurons, C57BL/6 mice brain slices (ex vivo), C57BL/6 mice (in vivo)	Reduced neuronal Cl^−^ accumulation and the development of LRDs in acute brain slices exposed to 0-Mg, acted to protect against PTZ-induced motor seizures, slowed the SE onset, and reduced the KA-induced seizures.	[156]
CLP257		KCC2	C57BL/6 mice hippocampal slices (ex vivo)	Reduced the duration and frequency of ictal-like epileptiform discharges (ILDs).	[150]
CLP290		KCC2	Tat^+^ and Tat^–^ mice (in vivo)	Restored phosphorylation of Ser940 and increased KCC2 membrane localization.	[154]
Indirect KCC2 modulators					
LM22A-4		TrkB	Naive P7 mouse pup brain slices (ex vivo)	Reduced postischemic neonatal seizure burdens at P7 and rescued ipsilateral KCC2 degradation.	[122]
ANA12		TrkB	CD1 mice (in vivo)	Rescued P7 or P10 post-ischemic KCC2 downregulation, improved phenobarbital-efficacy at P10.	[121]
WNK463 (orthosteric)		WNKs	C57BL/6 mice (in vivo)	Delayed onset of kainic acid-induced status epilepticus, less epileptiform EEG activity.	[86]
NEM		SPAK, PKC	Cultured cortical neurons or HEK293 cells (in vitro)	Increased the surface stability of KCC2 and reduces pThr1007, increased pSer940, thus enhanced KCC2 activity.	[53, 169]
KW-2449		FLT3	Cultured cortical neurons or mice hippocampal slices, or Mecp2 mutant mice	Increased the expression of KCC2, induced a significant hyperpolarizing shift in E_GABA_, and increased in the chloride extrusion rate in human RTT neurons, ameliorated disease-related behavioral pathologies in Mecp2 mutant mice.	[161]
KCC2 genetic modulator					
An adeno-associated virus-mediated CRISPRa system	CRISPRa	KCC2	Cultured cells, mouse hippocampal kindling model and mouse kainic acid-induced epilepticus model	Increased KCC2 expression both in cell culture and the targeted brain region in vivo, reduced the severity of hippocampal seizures and enhancing the anti-seizure effects of diazepam, and mitigated valproate-resistant spontaneous seizures.	[166]

*KCC2* K^+^-Cl^-^ cotransporter 2, *TrkB* tyrosine kinase receptor B, *WNKs* may include WNK1, WNK2, WNK3 and WNK4, *NEM* N-ethylmaleimide, *PKC* Protein kinase C, *SPAK* SPS1-related proline/alanine-rich kinase, *FLT3* tyrosine kinase 3, *MECP2* methyl CpG binding protein 2, *RTT* Rett syndrome, *LRDs* late recurrent discharges, *SE* status epilepticus, *KA* kainic acid, *PTZ* pentylenetetrazole; and *CRISPRa* CRISPR-mediated transcriptional activation.

### Idiopathic generalized epilepsy

Idiopathic Generalized Epilepsy (IGE), a form of generalized epilepsy suggested to be due to genetics, has also been associated with impaired Cl^-^ extrusion [34]. Kahle et al. utilized Sanger sequencing to screen for mutations in the cytoplasmic C-terminal of *SLC12A5* [44]. This targeted DNA sequencing method identified two heterozygous mutations: c.2855 G > A (R952H) and c.3145 C > T (R1049C) [44]. These variants demonstrated significantly impaired Cl^-^ extrusion capacity, as measured by a fluorescence-based assay (basal R_430/500_ decreased from 1.16 ± 0.05 to 0.87 ± 0.01 arbitrary units) [44, 49]. Consequently, these impaired Cl^-^ extrusion mechanisms resulted in cells with a higher basal intracellular Cl^-^ concentration and reduced hyperpolarizing response to glycine compared to the WT KCC2, as expected [44]. However, while R952H exhibited reduced KCC2 cell surface expression, R1049C did not [44]. This suggests that their different positions within the cytoplasmic C-terminus may have led to R952H compromising KCC2 function by decreasing cell surface expression, while R1049C reduced intrinsic KCC2 activity [44]. Therefore, this underscores the potential impact of the mutation’s localization on the epileptic phenotype.

Puskarjov et al. support findings that indicate R952H significantly reduces neuronal Cl^-^ extrusion, as measured by the somatodendritic E_GABA_ gradient (KCC2-R952H: ΔE_GABA_ = − 3.28 ± 0.33 mV/50 μm compared to KCC2-WT) [45]. Consequently, this results in a higher basal intracellular Cl^-^ concentration, which decreases the Cl^-^ driving force required to elicit a hyperpolarizing GABA response. These research findings are likely to have high validity because they utilize the soma-to-dendrite Cl^-^ gradient to measure Cl^-^ extrusion, allowing for the quantification of the ‘pure’ Cl^-^ extrusion capacity [50]. Furthermore, Puskarjov et al. demonstrated that R952H impaired KCC2’s ability to induce dendritic spine formation both in vivo and in vitro, suggesting that it could lead to desynchronization of excitability and promote seizures [45]. The link between the R952H mutation and increased neuronal excitability is further supported by a recent study on naked mole-rats with the same arginine to histidine point mutation, as found by Puskarjov et al. [45] and Kahle et al. [44]. Electrophysiology recordings showed that this variant resulted in naked mole-rats exhibiting reduced hyperpolarizing GABA signaling due to less efficient Cl^-^ extrusion [51]. This reinforces the importance of this specific position within KCC2’s regulatory region in limiting overexcitation.

Finally, despite exerting different effects on KCC2 trafficking, both R952H and R1049 variants exhibited decreased phosphorylation of the serine 940 (Ser940) residue. Typically, phosphorylated Ser940 enhances KCC2 activity and membrane stability [44, 52, 53]. This suggests that the reduced phosphorylation of KCC2-Ser940 may contribute to neuronal hyperexcitability in individuals with epilepsy, indicating that increasing KCC2-Ser940 phosphorylation could potentially alleviate this hyperexcitability. Consequently, it has been concluded that R952H is a susceptibility variant for IGE [44, 45]. Additionally, a novel KCC2 variant, V4731, has recently been discovered in Hungarian patients with IGE [41, 46]. The existing literature on epileptic patients with KCC2 mutations has emphasized the role of accumulated high intracellular Cl^-^ concentration resulting from reduced KCC2-dependent Cl^-^ extrusion, which may underlie the hyperexcitability observed in epilepsy. These findings indicate that KCC2 activation is necessary to prevent epilepsy’s pathophysiology. Overall, these genetic studies support the concept of enhancing KCC2 function by increasing its intrinsic trafficking ability and/or cell surface expression as potential therapeutic strategies for treating epilepsy.

Further functional studies focusing on the specific localization of the KCC2 mutations would provide valuable insights into how KCC2 can be enhanced to develop the most effective therapeutic strategy. Additionally, while Saitsu et al. [42] discussed the association between the identified mutations and their clinical features, further research is needed to evaluate how the localization of *SLC12A5* mutations affects epileptic severity, using a standardized rating scale. Conducting this research could help identify epileptic patients who are at a higher risk of developing severe epilepsy, thereby potentially influencing the treatment options available to them.

### Reduced KCC2 expression in epileptic patients

Research conducted on human brain slices has proposed that the generation of epileptic seizures involves an altered expression pattern between NKCC1 and KCC2, which determines the switch from hyperpolarizing to depolarizing GABA signaling [54–56]. Another study focusing on surgically resected brain specimens from temporal lobe tissue demonstrated reduced expression of KCC2 mRNA within the epileptic region of the hippocampal subiculum [19]. This reduction in KCC2 expression corresponded with greater expression in neurons that maintained hyperpolarization during interictal events, in contrast to depolarized cells [19]. However, it is important to note that this research only examined KCC2 expression and did not explore NKCC1 expression, which may lead to potential misunderstandings. Examining both KCC2 and NKCC1 expression is crucial to accurately understand the association between altered CCC expression and hyperexcitability involved in epileptogenesis since the NKCC1/KCC2 ratio plays a vital role in maintaining Cl^-^ homeostasis [11]. For instance, in the context of schizophrenia, an elevated expression ratio of NKCC1/KCC2 with reduced KCC2 levels has been shown to increase intracellular chloride concentration ([Cl^-^]_i_) [57–59]. Previous research investigating both KCC2 and NKCC1 expression levels within the hippocampal subiculum further supports the importance of studying both transporters [23]. In this study, human hippocampal tissue exhibited downregulated KCC2 and upregulated NKCC1, with the authors suggesting that the upregulation of NKCC1 also played a significant role in determining E_GABA_ [23]. Therefore, these findings suggest that altered NKCC1 expression is equally important as KCC2 in contributing to the hyperexcitability required for the generation of epileptic seizures.

Research on sclerosed hippocampi with mesial temporal lobe epilepsy (MTLE) further supports the concept of increased NKCC1 and decreased KCC1 expression contributing to epileptic activity [54, 56]. The sclerotic CA1 region of hippocampal sclerosis patients has demonstrated a lower degree of NKCC1/KCC2 colocalization than in non-sclerotic regions [56]. However, the usefulness of this study is limited due to potential inaccuracies in the data obtained from the control group. These inaccuracies may have arisen as the normal adult human brain tissue examined in this study was obtained post-mortem, potentially resulting in protein loss between death and Western blot analysis [54]. Despite this concern, Cai et al. found similar results using fresh hippocampal tissue, showing an increase in NKCC1 and a decrease in KCC2 expression in both the CA2 region and dentate gyrus of sclerosed hippocampi [54]. However, the change in NKCC1 expression elicited greater significance than KCC2 [54]. This greater change in NKCC1 expression than KCC2 suggests that NKCC1 is the main contributor to hyperexcitability underlying the patients’ epileptic seizures, as suggested previously by Palma et al. [23]. This therefore suggests that downregulating NKCC1 may be a more beneficial strategy for attenuating neuronal hyperexcitability in epileptic patients rather than targeting KCC2. However, both Cai et al. [54] and Munoz et al. [56] did not assess the differences in KCC2 expression between healthy and epileptic human hippocampi. The addition of a further analysis of hippocampal tissues from those with epilepsy would have been beneficial to determine the extent of the NKCC1 and KCC2 expression changes. In addition, none of the research discussed so far has correlated the extent of the changed KCC2 or NKCC1 expression with epileptic severity. A more recent study using a human dataset of 413 patients with tumor-associated epilepsy attempted to do this by using Kaplan-Meier analysis to determine the association between KCC2 expression and survival rate [55]. They found a strong correlation between decreased KCC2 gene expression and early death [55]. However, it has been suggested that Kaplan-Meier estimates can be misleading, and thus, this conclusion should be interpreted with caution [60]. Future research examining NKCC1 and KCC2 expression patterns in human epileptic tissue from patients with varying epileptic severity would be beneficial. This research would enable the correlation of the expression pattern of these CCCs with epileptic severity and could be used to determine the altered expression profile that increases an individual’s susceptibility to seizure generation and consequently the development of epilepsy.

Cortical dysplasia (CD) is a developmental abnormality of cortical organization and a common cause of drug-resistant epilepsy [61]. Munakata et al. investigated KCC2 expression in 18 CD specimens obtained during epilepsy surgery, comparing them with control sections [62]. The CD specimens consisted of 8 cases of focal CD (FCD) type I, 6 cases of FCD type II, and 4 cases of hemimegalencephaly (HME). In non-dysplastic cortex, KCC2 staining, by immunohistochemistry, was widespread in all layers. CD tissues showed lower staining intensity in cell bodies, while subcortical ectopic neurons exhibited dense intrasomatic staining. FCD type I displayed less intense KCC2 staining in cell bodies, aberrant giant pyramidal neurons showed reduced KCC2 staining, and immature neurons exhibited intrasomatic staining. FCD type II had dysmorphic neurons with intense intrasomatic staining and reduced KCC2 staining in neighboring neuropils. Balloon cells did not exhibit KCC2 staining. This study suggests that variations in KCC2 distribution may affect the ionic balance and epileptic activity within CD tissues. In another study, Han et al. analyzed surgical samples from 12 individuals with FCD and normal brain tissues from 6 autopsy cases without developmental abnormalities [63]. Patients’ ages ranged from 0.5 to 65 years, with 11 individuals above 14 years old and 7 individuals below 5 years old. The study identified two distinct patterns of abnormal GABAergic neuronal density in FCD: a "broad pattern" observed in 7 cases where both dysplastic and neighboring nondysplastic regions had reduced GABAergic neuron density, and a "restricted pattern" seen in the remaining cases where GABAergic neuron density decreased only in the dysplastic regions. These patterns were not linked to specific FCD subtypes. Notably, most FCD type II subjects (5 out of 7) exhibited intracytoplasmic retention of KCC2 in dysmorphic neurons, while this was not observed in FCD type I cases. Consequently, the study suggests that a "broad" GABAergic deficiency may indicate increased epilepsy susceptibility beyond the dysplastic region and that abnormal KCC2 distribution might contribute to seizure generation in FCD type II patients (4 out of 7 below 3 years old), but not in those with type I.

Gelastic seizures (GS) are rare epilepsy episodes of inappropriate laughter, often associated with hypothalamic hamartoma (HH). Wu et al. studied 93 neurons from 34 HH patients [64]. 76% were small (6–9 micrometer) and 24% were large (>20 micrometer). GABA_A_R activation had opposite effects on small and large HH neurons. Large neurons were depolarized/excited, while small neurons were hyperpolarized/inhibited. Large neurons had positive Cl^-^ equilibrium potentials, higher intracellular Cl^-^ concentrations, lower KCC2 expression, and an immature phenotype, indicating GABA_A_R-mediated excitation. These findings shed light on HH’s epileptogenicity, emphasizing GABA_A_R-mediated excitation as a contributing factor.

On the contrary, Karlocai et al. undertook a study to compare the levels of KCC2 expression in the hippocampus of TLE patients and control brain samples [65]. Through the use of Western blot analysis at the whole cell level and immunocytochemistry at the subcellular level, they discovered a widespread increase in KCC2 expression in individuals with epilepsy. Furthermore, the researchers performed parallel experiments on chronically epileptic mice and observed a similar distribution pattern of KCC2 [65]. There has been disagreement regarding whether the variances in KCC2 expression stem from variances in epileptic tissues, epileptic phases (such as acute, latent, chronic) [66], and the specific brain regions (for example, cortex, subiculum, hippocampus, cortical etc.) under investigation [19, 56, 67, 68]. Nevertheless, the techniques used (such as Western blot, immunofluorescence, immunohistochemistry, and RT-PCR) and the reliability of the antibodies or PCR primers employed can also influence the outcome of the research.

Nevertheless, spatially resolved studies have investigated the changes in KCC2 and NKCC1 induced by lesions, providing a clear understanding of whether precise, anatomically well-defined lesions can elicit spatially restricted, layer-specific alterations in the expression of these proteins. Turco et al. recently demonstrated that entorhinal denervation induces specific changes in the expression of KCC2 and NKCC1 in the molecular layers of the dentate gyrus, specifically the oml/mml layers [69]. Through the use of laser microdissection, microarray analysis, and RT-qPCR, they identified a decrease in KCC2 mRNA and reduced levels of KCC2 protein in denervated granule cell dendrites. Furthermore, they observed an increase in NKCC1 expression in reactive astrocytes within the oml/mml. This study suggests that the temporary decrease in KCC2 may facilitate GABAergic depolarization and denervation-induced spine loss, while the delayed recovery of KCC2 may contribute to compensatory spinogenesis.

### KCC2 rs2297201 gene polymorphism in epileptic patients

Dimitrijevic et al. conducted an analysis to investigate the association between KCC2 rs2297201 gene polymorphisms and the phenotypic expression of febrile seizures (FS) in a cohort of 112 patients diagnosed with FS, comparing them with a control group of healthy children [70]. The study revealed that the CT and TT genotypes, as well as the T allele of the rs2297201 polymorphism in the KCC2 gene, are risk factors for FS [70]. These findings provide initial evidence for the involvement of functional polymorphisms in the KCC2 gene in the development of febrile seizures.

### The impact of KCC2 in the viability and structure of developing and mature neurons

Kontou et al. investigated the impact of KCC2 on neuronal viability and structure by selectively eliminating its expression in developing and mature neurons [71]. Reduction of KCC2 expression in mature neurons promptly activated the extrinsic apoptotic pathway. Pharmacological inhibition of KCC2 in mature neurons induced apoptosis rapidly, independent of neuronal depolarization. In contrast, abolishing KCC2 expression in immature neurons did not significantly affect their subsequent development or structure. However, it did result in the cessation of hyperpolarizing GABA_A_R currents during postnatal development. These findings demonstrate that KCC2 plays a crucial role in preserving neuronal survival by limiting apoptosis in mature neurons, while having minimal influence on neuronal development or structure.

## Posttranslational regulatory mechanisms of KCC2

KCC2 functions are regulated by multiple posttranslational mechanisms, including phosphorylation, glycosylation, and ubiquitination, involving various pathways and feedback loops. These mechanisms enable precise regulation of chloride ion homeostasis in neurons and play a role in modulating inhibitory neurotransmission.

### Phospho-regulation of KCC2 activity

Several studies have shown a decrease in the functional expression of KCC2 in human epileptic patients [19, 23, 51, 54]. Epileptic patients have also exhibited reduced Cl^-^ extrusion capacity dependent on KCC2 activity [23, 56]. Identifying the regulatory mechanisms of KCC2 may lead to therapeutic strategies for increasing KCC2 activity to treat epileptic patients. The localization of key phospho-regulatory sites of KCC2 discussed is demonstrated in Fig. 2. The research findings from studies targeting the signaling pathways involved in the phospho-regulation of KCC2 are summarized in Table 3.

**Table 3:** Transgenic animal models used in modulating KCC2 activity via its direct and indirect modulation on epileptic seizure activity.

Target	Model	Method	Age	Findings	Reference
KCC2^a^	Rat hippocampal slices exposed to 4-AP^b^ *(*in vitro*)*	Electrophysiology	Adult	Enhanced KCC2 activity using CLP257 increased duration of ictal like discharges.	[151]
KCC2	P6-P7^c^ CLM1 mice hippocampal slices (in vitro)	Electrophysiology	Neonatal	Enhanced KCC2 activity using CLP257 decreased ictal like activity.	[150]
KCC2	Mouse with conditional knockout of KCC2 in Dlx5-lineage neurons (Dlx5 KCC2 cKO) (in vivo)	Electrophysiology		Loss of KCC2 caused occasional spontaneous seizures.	[159]
KCC2	A mouse line with 2–3-fold KCC2 overexpression occurs in pyramidal neurons (in vivo)	EEG^d^ monitoring	Adult	Enhanced KCC2 expression increased diazepam’s efficacy in stopping EEG seizures.	[160]
Preventing KCC2- Thr906/Thr1007 phosphorylation	Intraperitoneal injection of kainate in male KCC2-Thr906Ala/Thr1007Ala knock-in mice (in vivo)	EEG monitoring	Neonatal	Prevention of Thr906 and Thr1007 phosphorylation delayed the onset and severity of kainate induced seizures.	[80]
Constitutive KCC2-Thr906/Thr1007 phosphorylation	P15 transgenic mice with heterozygous phospho-mimetic mutations Thr1007Glu and Thr1007Glu exposed to flurothyl (in vivo)	Behavioral seizure recording	Adult	Constitutive phosphorylation at Thr906 and Thr1007 decreased latency to flurothyl induced seizures.	[76]
WNK^e^	Intraperitoneal injection of kainate in P7-P8 C75BL/6 mice (in vivo)	EEG monitoring	Neonatal	WNK inhibition by WNK463 increased latency to onset of SE^f^, reduced severity of kainate induced SE and prevented the development of diazepam resistance seizures.	[86]
Preventing KCC2-Ser940 phosphorylation	Intraperitoneal injection of kainate in P8 KCC2-Ser940Ala knock-in mice (in vivo)	EEG monitoring	Neonatal	Prevention of the KCC2-Ser940 phosphorylation increased severity of kainate induced seizures and resulted in lethality.	[103]
Preventing KCC2-Ser940 phosphorylation	Entorhinal cortex slices from P3-P5 KCC2-Ser940Ala knock-in mice exposed to 0-Mg^2+^ ACSF^g^ (in vitro)	Electrophysiology	Neonatal	Prevention of KCC2-Ser940 phosphorylation decreased the latency to SE.	[104]
TrkB^h^	Intrahippocampal injection of kainate in CD2 transgenic mice either overexpressing full length TrkB receptor or the truncated form (TrkB-T1) (in vivo)	EEG monitoring	Adult	TrkB receptor overexpression decreased the duration of kainate induced seizures, whilst reduced TrkB signaling (TrkB-T1) delayed kainate induced seizures.	[115]
TrkB and BDNF^i^	Electrical kindling in transgenic mice exhibiting BDNF or TrkB deletion (in vivo)	Behavioral seizure scoring and EEG monitoring	Adult	Conditional deletion of TrkB prevented the development of electrical kindling. Deletion of BDNF partially inhibitied electrical kindling.	[116]

^a^KCC2, K^+^-Cl^-^−cotransporter 2.

^b^4-AP, 4-Aminopyridine.

^c^P6-P7, between postnatal day 6 and postnatal day 7.

^d^EEG, Electroencephalogram.

^e^WNK, Lysine-deficient protein kinase.

^f^SE, Status epilepticus.

^g^ACSF, Artificial cerebrospinal fluid.

^h^TrkB, Tropomyosin receptor kinase B.

^i^BDNF, Brain-derived neurotrophic factor.

#### WNK-regulated SPAK/OSR1 kinases-dependent KCC2 phosphorylation

**Fig. 3:**
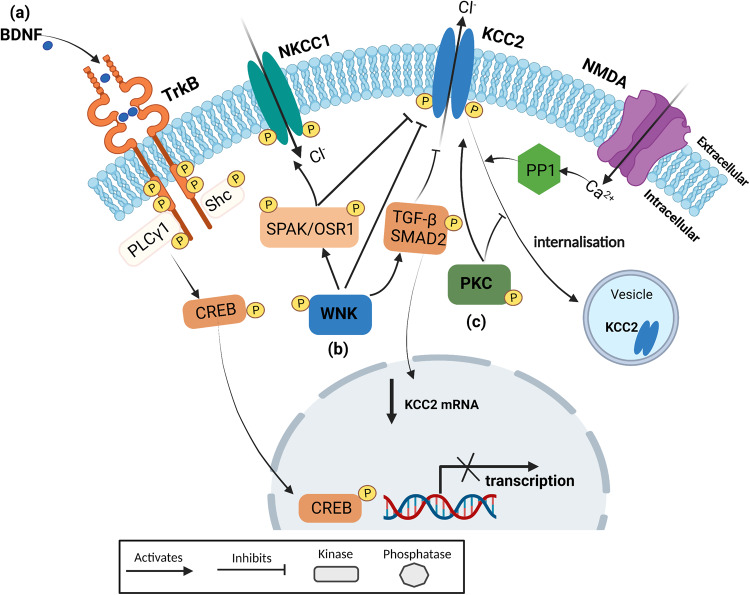
Regulation mechanisms of KCC2 in mature neurons. **a** BDNF-TrkB Signaling: Binding of BDNF to its TrkB receptor leads to autophosphorylation of tyrosine residues within the receptor. This creates docking sites for the adaptor protein Shc and phospholipase Cγ (PLCγ), activating second messengers that stimulate a downstream cascade, resulting in the phosphorylation and activation of CREB. CREB binds to the transcriptional machinery within the nucleus to control gene expression, leading to reduced KCC2 gene transcription in mature neurons. **b** WNK-SPAK/OSR1 Signaling: WNK-SPAK/OSR1 signaling regulates the activity of NKCC1 and KCC2 through phosphorylation. WNK phosphorylates and activates SPAK/OSR1. The activated WNK-SPAK/OSR1 signaling pathway phosphorylates NKCC1 at Thr203, Thr207, and Thr212 and KCC2 at Thr906 and Thr1007 residues, resulting in their activation and inhibition, respectively. WNK1 collaborates with TGF-β and Smad2 in KCC2 expression and phosphorylation at Thr1007. **c** PKC and PP1 Regulation: PKC and PP1 have reciprocal roles in regulating KCC2 activity. PKC phosphorylates KCC2 at the Ser940 residue, stabilizing it at the neuronal cell surface membrane. Conversely, PP1, which is activated by high NMDA receptor activity, stimulates the internalization of KCC2, consequently reducing neuronal KCC2 activity. The diagram was created using BioRender.com. BDNF brain-derived neurotrophic factor, TrkB tropomyosin-related kinase receptor type B, PLCγ phospholipase C gamma 1, CREB cAMP response element-binding protein, NKCC1 Na^+^-K^+^-Cl^-^ cotransporter 1, KCC2 K^+^-Cl^-^ cotransporter 2, WNK With-No-Lysine (K) kinases, SPAK SPS1-related proline/alanine-rich kinase, OSR1 oxidative stress-responsive kinase 1, TGF-β2 transforming growth factor beta 2, PKC protein kinase C, PP1 protein phosphatase 1, and NMDA N-Nitrosodimethylamine.

Phosphorylation of NKCC1 and KCC2 is a crucial post-translational regulatory mechanism controlling the activity and expression of these CCCs at the cell surface membrane, as illustrated in Fig. 3. The With-No-Lysine (K) kinases (WNKs) regulating SPS/Ste20-related proline-alanine-rich kinase (SPAK)/oxidative stress-responsive kinase 1 (OSR1) pathway has been shown to reciprocally regulate NKCC1 and KCC2 activity, resulting in inhibition and activation, respectively, to tightly coordinate Cl^-^ homeostasis [39, 72]. WNK (lysine-deficient protein kinase), mainly WNK1, WNK3, and WNK4 within the brain, indirectly regulate KCC2 and NKCC1 activity [72, 73]. Low intracellular Cl^-^ concentration activates WNK to directly phosphorylate the two kinases, SPAK (SPS1-related proline/alanine-rich kinase) and OSR1 (oxidative stress-responsive kinase 1), on their threonine residues within the T-loop motif and serine residues within the S-motif [73, 74]. This stimulates the activation of SPAK/OSR1 kinase activity, resulting in the phosphorylation of KCC2 at various serine and threonine residues [73, 75, 76]. The phosphorylation of threonine 906 (Thr906) and threonine 1007 (Thr1007) residues of KCC2 by the WNK-SPAK/OSR1 kinase complex manifests inhibitory properties [39, 75]. The extent of WNK phospho-regulation of KCC2 is altered during development, with a decrease in KCC2 phosphorylation as neuronal development progresses [75, 77].

Most of the research conducted on WNK-dependent phospho-regulation of CCCs has focused on renal epithelial tissue in the context of studying hypertension, with limited literature discussing this regulation in brain tissue [39, 73, 78]. However, Friedel et al. demonstrated that WNK1 stimulates the phosphorylation of KCC2 at Thr906 and Thr1007, resulting in KCC2 inhibition in rat cultured hippocampal neurons [75]. In our study, we observed increased phosphorylation of WNK1, SPAK, and OSR1 occurs in low intracellular Cl^-^ concentrations when GABA_A_R activation was prevented using gabazine [72]. Additionally, expression assays revealed that gabazine reduced KCC2 cell surface expression by approximately 22% (*P*  <  0.01, exact expression levels not stated), increased KCC2 lateral diffusion, and reduced KCC2 clustering, suggesting that phosphorylation of Thr1007 and Thr906 decreases KCC2 cell surface membrane stability. More recently, researchers have studied the effect of constitutive phosphorylation at Thr906 and Thr1007 on epileptic activity. To investigate this further, knock-in mice expressing homozygous dual glutamate (E) substitutions at Thr906/Thr1007 (“*KCC2*^*E/E*^”) were developed, resulting in constitutive phospho-mimetic inhibition, compromised neuronal Cl^-^ extrusion, and early post-natal death from respiratory arrest [79]. Heterozygous *KCC2*^*E/E*^ mice exhibited altered GABAergic inhibition, increased susceptibility to epileptic seizures, and other neurodevelopmental defects. Furthermore, hippocampal neurons in the heterozygous *KCC2*^*E/+*^ mice showed a delay in the developmental hyperpolarizing GABA shift, and transgenic mice displayed increased seizure susceptibility at P15, along with deficits in social interactions [76]. In contrast, Moore and colleagues took the opposite approach and generated a transgenic mouse strain with homozygous dual alanine (A) substitutions at Thr906/Thr1007 (“*KCC2*^*A/A*^”), preventing phospho-dependent inactivation [80]. These *KCC2*^*A/A*^ mice exhibited increased basal neuronal Cl^-^ extrusion and reduced drug-induced epileptic activity [80]. These findings suggest that overactivity of WNK-SPAK/OSR1, leading to impaired KCC2 function, may contribute to the pathogenicity of epilepsy and vice versa. Taken together, these findings support the concept of WNK1 inhibition as a promising strategy to increase KCC2-dependent Cl^-^ extrusion, lower intracellular Cl^-^ concentration, restore GABAergic inhibition, and consequently reduce seizure activity. However, to the best of our knowledge, no research has correlated the extent of KCC2 phosphorylation at Thr906 and Thr1007 with KCC2 activity. Future functional assays examining this relationship would be beneficial in determining the amount of WNK or SPAK activation (measured by their phosphorylation levels) required to elicit excitatory GABAergic signaling. Furthermore, genetic studies on epileptic patients are needed to discover potential genetic variants in WNK proteins that contribute to overactive WNK-SPAK/OSR1 signaling, in order to determine if this is a genetic risk factor for epilepsy. Indeed, our recent study utilized exome sequencing and variant validation, which led to the identification of six rare single nucleotide variants (SNVs) in *WNK3* from six unrelated families. The affected individuals displayed intellectual disability phenotypes, with varying presence of epilepsy and structural brain defects [81]. This is the first reported association of WNK3 mutation with human epilepsy. Biochemical assays conducted on three WNK3 pathogenic missense variants (p.(Pro204Arg), p.(Leu300Ser), and p.(Glu607Val)) indicated that all three variants induce WNK3 degradation and impair the regulatory phosphorylation of KCC2 [81]. However, further investigation is necessary to understand the impact of these variants on KCC2 function and the GABA excitatory-inhibitory transition in in vivo models.

The literature discussed thus far indicates that increased phosphorylation at Thr906 and Thr1007 contributes to hyperexcitability. However, it has also been demonstrated that epileptic activity itself stimulates KCC2 phosphorylation at these threonine residues [72, 76, 80, 82]. In a study by Yang et al., the post-epileptic expression profile of SPAK was examined in the hippocampus of mice affected by pilocarpine-induced status epilepticus (PISE), providing the first research linking SPAK expression to epilepsy [82]. It was found that SPAK mRNA and protein levels were significantly increased until 45 days after PISE induction, with the peak expression observed 14 days post PISE [82]. This aligns with a significant increase in KCC2-Thr1007 phosphorylation (150% ± 17% of WT littermates) in hippocampal tissue following kainate-induced seizures [80]. These findings suggest that SPAK expression increases as a result of both pilocarpine-induced and kainate-induced epileptic activity, shedding light on the involvement of the WNK-SPAK/OSR1 pathway in epilepsy. However, it is important to note that the generalizability of these findings is limited as the study employed a model of epilepsy instead of studying epileptic tissue directly [83, 84]. Future research should focus on investigating the changes in WNK expression patterns in brain tissue of epileptic patients to gain a more direct understanding of the role of WNK-SPAK/OSR1 pathway in human epilepsy.

WNK has been shown to decrease KCC2 activity, which could contribute to the elevated intracellular Cl^-^ concentrations observed in epileptic activity. Consequently, WNK-SPAK/OSR1 kinase inhibitors have been proposed as an indirect approach to modulate intracellular Cl^-^ levels by inhibiting NKCC1 and activating KCC2. These inhibitors would effectively reduce Cl^-^ levels, thereby restoring inhibitory GABA signaling. Previous studies have demonstrated that WNK inhibition enhances KCC2 activity [72, 75]. In our previous research, we transfected mouse neuroblastoma neuro-2a (N2a) cells with WNK-AS, a chemically genetically altered WNK variant susceptible to inhibition by protein phosphatase 1 (PP1) [75]. Inhibiting WNK1 resulted in enhanced KCC2 activity, as evidenced by a faster fluorescence recovery in response to GABA_A_R activation, along with a decrease in Thr906 and Thr1007 phosphorylation[75]. This study is particularly valuable as it employed a method of WNK1 inhibition that mimics a potential therapeutic agent. Additionally, the use of N2a cells, which are known to express low levels of KCC2 [85], offers the advantage of simulating the reduced KCC2 expression observed in epileptic patients [19, 54]. Furthermore, genetic silencing of WNK1 using specific short hairpin RNAs (shRNA) prevented the increase in KCC2 diffusion, demonstrating the effectiveness of WNK inhibition in enhancing KCC2 membrane stability [72]. The increase in KCC2 activity resulting from WNK1 inhibition holds promise for promoting inhibitory GABAergic signaling [75, 86]. Both WNK inhibition through genetic silencing and a selective WNK kinase inhibitor (WNK463) (Table 2) induce a significant hyperpolarizing shift in E_GABA_ of approximately 15 mV and 11 mV, respectively, in immature cortical rat neurons (pre WNK463 treatment neuronal baseline E_GABA_: −67 ± 4 mV; post WNK463 treatment E_GABA_: −78 ± 4 mV, *P* < 0.0001; and genetic silencing baseline E_GABA_: −57.9 ± 1.5 mV) [75, 86]. However, it is worth noting that mature cortical neurons did not exhibit a significant negative shift in E_GABA_ (an approximate 8 mV shift) [75]. This discrepancy may be attributed to mature neurons already having a more hyperpolarized E_GABA_ value compared to immature neurons, suggesting that a more depolarized E_GABA_ is required [75]. Additionally, the higher basal E_GABA_ value in mature neurons may explain why WNK463 produced a more pronounced negative shift in E_GABA_ compared to WNK genetic silencing. Further research is needed to evaluate the effectiveness of WNK inhibition in individuals with normal phosphorylation patterns of KCC2.

The effect of WNK inhibition on seizure susceptibility needs to be assessed to determine its potential as a strategy for treating seizures in epileptic patients. Some evidence suggests that WNK inhibition could be an effective anticonvulsant approach in vivo [76, 80, 86]. Lee et al. conducted a study where an orally bioavailable WNK inhibitor, WNK463, was administered to C57BL/6 mice at postnatal day 7–8 (P7-P8) [86]. The intrahippocampal administration of WNK463 delayed the onset of status epilepticus and reduced the severity of kainate acid (KA)-induced status epilepticus [86]. These findings are consistent with earlier research that utilized threonine 906/1007 sites knock-in mice (*KCC2*^*A/A*^ mice) to prevent WNK phospho-dependent KCC2 inhibition [80]. The *KCC2*^*A/A*^ mice also demonstrated a reduced severity of KA-induced seizures using similar EEG recording and power spectra analysis methods [80]. However, unlike pharmacologically inhibited WNK1, *KCC2*^*A/A*^ mice experienced a significant delay in the onset of the first KA-induced seizure rather than solely delaying status epilepticus [80, 86]. Despite these differences, this research indicates that inhibiting Thr906 and Thr1007 phosphorylation to increase KCC2 activity is sufficient to attenuate the development and severity of epileptic activity. It is worth noting that pharmacological WNK inhibition did not reduce the mortality rate in mice following KA-induced status epilepticus, whereas a reduced number of *Kcc2*^*A/A*^ mice died compared to WT mice [80, 86]. The contrasting findings between Moore et al. [80] and Lee et al. [86] are likely due to the different methods of WNK inhibition employed. Furthermore, these findings suggest that pharmacological WNK inhibition may not improve survival in the KA-induced epileptic model. However, it should be considered that kainate is a potent chemoconvulsant, and therefore the reduction in KCC2 activity induced by kainate might be greater than what is observed in human epileptic tissue [80]. This suggests that attenuating seizures through WNK inhibition may be more effective in epileptic patients. Additionally, it is important to note that WNK inhibition does not alter KCC2 surface expression, indicating that the prevention of Thr906 and Thr1007 phosphorylation increases the functional activity of KCC2 [80, 86]. However, this finding contradicts previous research conducted by Conway et al., who demonstrated that N-ethylmaleimide (NEM), an organic compound that inhibits Thr1007 phosphorylation by WNK inhibition (Table 2), actually increases KCC2 cell surface levels [53]. Nevertheless, this increase in surface levels may be due to NEM increasing phosphorylation of Ser940, which could be the underlying cause. Furthermore, NEM may also affect other KCC2 sites such as Ser31, Thr34, and Ser932 [87], as well as Thr934 and Ser937 [88]. Consequently, future functional studies are required to determine the exact mechanism underlying the enhancement of KCC2 activity through the prevention of Thr906 and Thr1007 phosphorylation.

Finally, WNK463 has demonstrated its ability to prevent the development of diazepam-resistant seizures, as evidenced by reduced epileptic EEG activity in mice treated with diazepam-resistant drugs [86]. This suggests an additional therapeutic application of pharmacological WNK inhibition as an adjunct strategy for patients with diazepam-refractory seizures. However, further research is necessary to investigate the efficacy of WNK inhibition on different animal models of drug-refractory epilepsy and validate these findings. It is important to note that there are four isoforms of WNKs expressed in various tissues, and adverse effects have been observed with WNK463, which hinder its further development [89]. Therefore, dedicated chemical efforts are required to develop more selective and specific inhibitors targeting WNK1. Alternatively, it may be worthwhile to explore the use of effective SPAK kinase inhibitors with good brain penetration, such as ZT-1a [90], in animal models of epilepsy.

#### WNK1 collaborates with TGF-β2 and Smad2 in KCC2 expression and phosphorylation

Roussa et al. previously identified the signaling pathway connecting transforming growth factor beta 2 (TGF-β2) to cAMP-response-element-binding protein (CREB) and Ras-associated binding protein 11b (Rab11b) as the fundamental mechanism behind TGF-β2-induced trafficking and functional activation of KCC2 [91]. TGF-β2 enhanced the colocalization and interaction between KCC2 and Rab11b, and impairing CREB1 or Rab11b hindered TGF-β2-mediated trafficking, surface expression, and functionality of KCC2. In a recent study, Rigkou et al. examined the effects of TGF-β2 on KCC2 during neuronal maturation [92]. They found that inhibiting TGF-β/activin signaling decreased KCC2 mRNA expression in immature neurons. TGF-β2 deficiency in mice resulted in reduced KCC2 expression, AP2β transcription factor, and KCC2 protein in the forebrain. The binding of AP2β to the KCC2 promoter was absent in TGF-β2-deficient mice. Additionally, TGF-β2 deficiency led to increased KCC2 phosphorylation at T1007 and decreased membrane KCC2 in pre-Bötzinger-complex neurons. These effects were rescued with exogenous TGF-β2. The study highlights the role of TGF-β2 in regulating KCC2 transcription in immature neurons, potentially acting upstream of AP2β, and contributing to KCC2 dephosphorylation at Thr1007 during development. TGF-β2 has diverse effects on KCC2 during neuronal maturation, providing insights into TGF-β2’s regulation of KCC2 expression, posttranslational modification, and surface expression. The study proposes that TGF-β2 is a significant regulator of KCC2 with implications for pathophysiological conditions. Interestingly, Cobb and colleagues previously discovered that WNK1 and WNK4 directly phosphorylate Smad2 [93], and recently found the functional interactions of TGF-β receptors with WNK1/OSR1 kinases [94]. Knockdown of WNK1 in HeLa cells using small interfering RNA reduces Smad2 protein expression due to down-regulation of Smad2 transcription. Conversely, WNK1 depletion leads to nuclear accumulation of phosphorylated Smad2, enhancing Smad-mediated transcriptional responses. Moreover, WNK1 small interfering RNA cells exhibit increased TGF-β-induced target gene transcripts. These findings highlight WNK1 as a dual modulator of TGF-β-Smad2 signaling pathways for TGF-β-regulated functions on KCC2. Bar-Klein et al. previously demonstrated that losartan, an angiotensin II type 1 receptor antagonist known to block peripheral TGF-β signaling, effectively inhibits albumin-induced TGF-β activation in the brain [95]. This suggests that blocking the TGF-β pathway may be beneficial in preventing epilepsy. However, further studies should explore the role of TGF-β signaling and KCC2 in epileptogenesis.

#### Thr1007 site phosphorylation dependent ubiquitin degradation of KCC2 by ubiquitin ligase Fbxl4

F-box and leucine-rich repeat protein 4 (Fbxl4) was previously identified as a clock output molecule that controls sleep by facilitating the rhythmic degradation of the GABA_A_R [96]. Recently, Hu et al. highlighted that the recovery of consciousness is not a passive process but an active one [97]. According to their findings, the activation of specific neural circuits could be associated with the restoration of consciousness and may hold significant importance in facilitating it. In their study, Hu et al. discovered a vital mechanism for actively recovering consciousness, involving Fbxl4-mediated ubiquitin degradation of KCC2 and phosphorylation specifically at the Thr1007 site, rather than the Thr906 site. This crucial process takes place in the ventral posteromedial nucleus (VPM) brain region [97]. Lowered total KCC2 levels and heightened phosphorylation at the Thr1007 site observed during the minimum responsive state caused a reduction in KCC2 activity, resulting in higher levels of [Cl^-^]_i_. This promoted Cl^-^ output driven by GABA and consequently led to depolarization mediated by GABA_A_ receptors in VPM neurons. By specifically inhibiting the phosphorylation of the KCC2 Thr1007 site in the VPM brain region of mice while they were under general anesthesia, the level of KCC2 protein increased. This led to a further prolongation of the loss of consciousness and intensified the anesthetic effect. These findings indicate that blocking this effect through KCC2 antagonists shows promise as a potential therapeutic approach. The WNK-SPAK/OSR1 kinases directly regulate the KCC2 Thr1007 site, but the kinase regulator of Thr906 remains unknown. Therefore, it is highly likely that the WNK-SPAK/OSR1 signaling pathway is involved in the active recovery of consciousness following propofol anesthesia [98]. Exploring this pathway in future studies would be worthwhile. Moreover, Hu et al. made an observation that animals under anesthesia displayed persistent tremors resembling seizures in their front limbs, lasting approximately 10–40 min prior to regaining consciousness. This implies a potential connection between the degradation of KCC2 through ubiquitin and the occurrence of anesthesia-induced epilepsy. Nevertheless, the precise mechanisms underlying the regulation of KCC2 expression through ubiquitination remain inadequately comprehended, necessitating further investigation.

#### PKC-dependent KCC2 phosphorylation

Another mechanism of phospho-regulation of KCC2 involves the phosphorylation of Ser940 within its C-terminal domain (Fig. 2). This phosphorylation is mediated by protein kinase C (PKC), leading to enhanced KCC2 activity [99, 100]. The increased activity of KCC2 is attributed to its improved cell surface stability, which reduces endocytosis and decreases the rate of internalization [99, 100]. Conversely, the dephosphorylation of KCC2-Ser940 is stimulated by high levels of glutamate and increased NMDA receptor (N-Methyl-D-Aspartate receptor) activity, which activates PP1 to remove the phosphate group from this residue [100]. The regulatory mechanism of KCC2 involving KCC2-Ser940 phosphorylation and dephosphorylation is illustrated in Fig. 3.

Research has demonstrated the importance of KCC2-Ser940 phosphorylation in maintaining GABAergic inhibitory signaling. Dephosphorylation of Ser940 has been shown to coincide with reduced GABAergic inhibition and hyperexcitability [100, 101]. In vitro studies have shown that preventing the dephosphorylation of Ser940 through pharmacological inhibition of PP1 attenuates the decrease in KCC2 cell surface expression induced by glutamate [100]. Moreover, this PP1 inhibition has been shown to be sufficient in maintaining GABAergic inhibition [100]. However, it should be noted that these findings were obtained using cultured rat hippocampal neurons, which only serve as a model for adult neurons [100]. Therefore, for greater usefulness, it would be necessary to measure the effect of PP1 inhibition in vivo using an epilepsy model. Despite this limitation, these findings align with later research conducted on a glioma xenograft model of tumor-associated epilepsy. In this study, the dephosphorylation of KCC2-Ser940 was associated with increased depolarizing GABAergic signaling and spontaneous seizure generation in vivo [101]. This suggests that the dephosphorylation of Ser940 is involved in the pathophysiology of tumor-associated epilepsy. However, the researchers did not examine whether preventing the dephosphorylation of Ser940 decreased seizure activity, as their focus was on elucidating the mechanism behind the epileptiform activity in glioma mice [101]. Nevertheless, they did demonstrate that inhibiting the intracellular accumulation of Cl^-^ through the presence of bumetanide (an NKCC1 blocker) reduced seizure susceptibility in glioma-implanted mice [101]. This indicates that modulation of KCC2 could also be effective, as bumetanide restores Cl^-^ homeostasis and acts as an effective anticonvulsant in this model. Given the increase in glutamatergic excitatory signaling associated with epileptic seizures, this literature suggests that a therapeutic agent inhibiting PP1 could be an effective treatment for tumor-associated epilepsy. Such an agent could decrease the rate of KCC2-Ser940 dephosphorylation and limit the excitatory GABAergic signaling associated with this dephosphorylation [102].

Phosphorylation of KCC2-Ser940 is crucial in preventing the development of severe epileptiform activity [103, 104]. To investigate the role of KCC2-Ser940 phosphorylation in seizure severity, Silayeva et al. generated knock-in mice with a KCC2 point mutation where Ser940 was mutated to alanine (KCC2-Ser940Ala) to prevent Ser940 phosphorylation [103]. The study found that when these mice were exposed to kainate, they exhibited greater seizure power (measured through EEG power spectra) compared to wild-type (WT) mice during status epilepticus, indicating increased seizure severity [103]. Furthermore, the increased seizure severity was confirmed by the rapid death of Ser940Ala mice after status epilepticus induction with kainate, while no lethality was observed in WT mice during EEG monitoring [103]. While these findings emphasize the importance of Ser940 phosphorylation in limiting seizure severity, they only indicate the impact of the loss of Ser940 phosphorylation on extreme epileptiform activity (status epilepticus). However, in acute entorhinal-hippocampal slices of KCC2-Ser940Ala mutant mice under 0-Mg^2+^ conditions (a less extreme in vitro epilepsy model), a lack of termination of seizure-like events and faster progression to status epilepticus were observed [103, 104]. This demonstrates that Ser940 phosphorylation is a critical phospho-regulatory mechanism that restricts the progression of seizures into status epilepticus. Nonetheless, further research is needed to investigate the significance of Ser940 phosphorylation in limiting less extreme epileptic activity. This is crucial as treatments that effectively attenuate less extreme seizure activity would benefit a broader range of epileptic patients. Currently, the literature only suggests that enhancing KCC2-Ser940 phosphorylation would be beneficial for epileptic patients experiencing severe seizures. Additionally, a decrease in Ser940 phosphorylation has been identified in patients with idiopathic generalized epilepsy, specifically those with R952H and R1049 KCC2 mutations [44]. This highlights the involvement of decreased KCC2-Ser940 phosphorylation in the pathophysiology of epilepsy. However, further research is required to establish the underlying mechanism by which these KCC2 mutations lead to a decrease in Ser940 phosphorylation. For instance, investigating the intrinsic ability of KCC2-Ser940 to be phosphorylated in a R952H mutant mouse would be necessary. This research would help determine if indirectly increasing KCC2 function by inhibiting PP1 or enhancing PKC would be effective in preventing epileptic seizures in individuals carrying this mutation. Furthermore, studies utilizing human epileptic tissue for in vitro functional assessments are needed to evaluate the efficacy of PP1 inhibitors and/or PKC activators in enhancing KCC2-Ser940 phosphorylation and reducing seizure severity.

#### Src family kinase-dependent KCC2 phosphorylation

An earlier study by Kelsch et al. found that the activation of KCC2 function requires the activity of tyrosine kinases, including cytosolic protein tyrosine kinase and C-Src tyrosine kinase (c-Src). They applied two membrane-permeable protein tyrosine kinase inhibitors, genistein or lavendustin A, to mediate the developmental switch of GABAergic responses to hyperpolarizing inhibition [105]. Impairment of Zn^2+^-induced E_GABA_ depolarization in cultured hippocampal neurons was observed in the presence of Src kinases inhibitor (PP2) or the tropomyosin-related kinase receptor type B (TrkB) inhibitor (K252A) [106]. Rapidly decreased tyrosine phosphorylation of KCC2 in rat hippocampal neurons was observed under conditions of oxidative stress (H_2_O_2_), induction of seizure activity (BDNF), and hyperexcitability (0 Mg^2+^) [107]. Reduced KCC2 tyrosine phosphorylation was also found to be correlated with a decrease in [Cl^-^]_i_ and a reduction in transport activity [107]. However, opposite results were observed in another study, showing increased KCC2 tyrosine phosphorylation at Y903/Y1087 in rat brain slices after pilocarpine-induced status epilepticus or in primary neuronal cultures treated with carbachol [108]. Due to discrepancies in these results, it is therefore not certain whether tyrosine phosphorylation of KCC2 plays an important role in functional transport activity. Further studies to clarify the importance of KCC2 tyrosine phosphorylation have been suggested in a previous review by Medina et al. [50].

### Other mechanisms in regulating KCC2

#### Trophic factors

BDNF is one of the most extensively studied trophic factors that regulate KCC2 activity [50]. BDNF binds to its receptor TrkB with high affinity [50], and upon binding, intracellular cascades are activated, leading to altered gene transcription that regulates KCC2 activity (Fig. 3) [109–111]. Aguado et al. first discovered the role of BDNF in regulating KCC2 expression by demonstrating that transgenic overexpression of BDNF significantly increased KCC2 mRNA levels in developing neurons [112]. However, it was later found that this upregulation only occurs in immature neurons, and in contrast, BDNF downregulates KCC2 expression in mature adult neurons [109, 112–114]. Figure 3 summarizes the mechanisms by which BDNF-TrkB signaling regulates KCC2 activity. The research findings from studies targeting BDNF-TrkB signaling to modulate KCC2 activity are summarized in Table 3.

Seizure activity elevates the levels of BDNF and TrkB, as neuronal activity has been shown to stimulate the release of BDNF and subsequently activate BDNF-TrkB signaling [109, 110, 115]. This suggests that BDNF-TrkB signaling may be involved in the downregulation of KCC2 in epileptic patients. However, despite the increase in BDNF associated with heightened neuronal activity, animal models have demonstrated that this elevated BDNF also promotes epileptic activity in vivo [115–117]. The pro-epileptic effect of BDNF has been demonstrated using a transgenic mouse model that either overexpressed the full-length (normal) TrkB receptor or overexpressed a truncated form, mimicking reduced BDNF-TrkB signaling [115]. This genetic modification approach allows for establishing the causal role of BDNF and TrkB in epileptic activity without pharmacologically inhibiting TrkB receptor activation. Transgenic mice with enhanced TrkB signaling showed faster development of epileptogenesis compared to delayed epileptogenesis in mutants with reduced TrkB signaling [115]. However, since TrkB activity is only reduced and not abolished in the transgenic mice, it suggests that pharmacological blockade of BDNF-TrkB signaling would lead to even greater disruption of epileptogenesis and thus has the potential to prevent epileptic development in children at risk of MTLE [115]. These findings are consistent with previous observations in homozygous TrkB knockout mice in the electrical kindling model [116]. He et al. demonstrated that the deletion of TrkB receptors and, hence, prevention of TrkB activation, prevented epileptogenesis, while in BDNF^-/-^ mice where TrkB activation was not fully inhibited, only modest impairment of epileptogenesis was observed [116]. Subsequent research investigating the TrkB-dependent activation of phospholipase C gamma 1 indicated that PLCγ1 signaling may underlie this epileptogenesis [118]. Therefore, it is proposed that TrkB activation is crucial for the development of MTLE.

The role of TrkB activation in epileptogenesis, as discussed previously, suggests that preventing the activation of BDNF-TrkB signaling or its downstream cascade involving PLCγ1 could potentially prevent the development of epilepsy. This concept has led to the development of ANA12 (Table 2), a low-molecular-weight TrkB ligand [119]. ANA12 binds non-competitively to the extracellular domain of the TrkB receptor to selectively block BDNF-TrkB signaling [119]. ANA12 has shown effectiveness in enhancing the efficacy of phenobarbital (PB), which is commonly used to treat neonatal seizures [120, 121]. The combination of PB and ANA12 has been found to successfully alleviate PB-resistant seizures in CD-1 mice, with ANA12 doses as low as 2.5 mg/kg [120, 121]. However, it should be noted that the study conducted by Kang et al. [120] reported a 20% greater improvement in PB efficacy compared to the later study by Carter et al. [121], even though both studies used the same ANA12 concentration (2.5 mg/kg). The measured values of ANA12 + PB suppression of seizures were −62% and −42% ±5.3% according to Carter et al. [121] and Kang et al. [120], respectively. The enhanced seizure suppression observed in the study by Carter et al. [121] may be attributed to a greater ischemia-induced downregulation of KCC2 in the ipsilateral hemisphere of the mice. Additionally, ANA12 demonstrated the ability to prevent post-ischemic downregulation of KCC2, as evidenced by rescuing both KCC2 and phosphorylated KCC2-Ser940 [121]. This research collectively underscores the therapeutic benefits of ANA12 by preventing the decrease in KCC2 activity and maintaining a low intracellular Cl^-^ concentration. Interestingly, contrary to the aforementioned research, more recent studies have shown that TrkB agonists (LM22A-4, HIOC, and deoxygedunin) can prevent post-ischemic downregulation of KCC2 and rescue PB-refractory seizures in the same neonatal mouse model by inhibiting BDNF-TrkB signaling [122]. This alternative approach to prevent BDNF-TrkB signaling further emphasizes the role of TrkB in neonatal seizure susceptibility. These findings also propose an alternative therapeutic strategy for treating patients with PB-refractory seizures, which should be investigated in future research.

Regarding ANA12, it should be considered a promising therapeutic agent due to its ability to penetrate the blood-brain barrier, thus addressing the limitations of the previously investigated NKCC1 antagonist bumetanide [119, 123]. Consequently, ANA12 may exhibit superior therapeutic efficacy in epileptic patients. Furthermore, when systematically administered to the brains of adult mice, ANA12 was found to inhibit TrkB activity without affecting neuronal survival or inducing significant adverse effects [119]. However, despite these promising findings, concerns have been raised about the selectivity of ANA12 due to the lack of comprehensive receptor interaction screening [124]. Conducting such screening is necessary before clinical implementation. Additionally, it should be noted that ANA12 was unable to prevent the occurrence of ischemic seizures or mitigate post-ischemic degradation of KCC2 in the absence of PB [120]. Therefore, the usefulness of ANA12 as an adjunct therapeutic agent for individuals with PB-resistant seizures appears limited. Further research and investigations are needed to better understand ANA12’s selectivity and its potential benefits in treating specific seizure conditions.

#### KCC2 glycosylation regulation

KCC2 possesses a total of six N-glycosylation sites located within the extracellular loop connecting TM5 and TM6 (Fig. 2). Notably, glycosylation of KCC2 has been observed in neurons at various developmental stages, encompassing both immature and mature states [125]. The glycosylation of KCC2 has been shown to significantly impact transporter function, as well as its relationship to KCC2 membrane trafficking. In a study conducted by Stödberg et al., patients with EIMFS were examined, focusing on loss-of-function mutations in the SLC12A5 gene [43]. The study revealed that both the expression and glycosylation of the KCC2 protein were diminished, resulting in a decrease in KCC2 activity and subsequent reduction in synaptic inhibition. Puskarjov et al. investigated the role of BDNF in the regulation of KCC2 glycosylation during seizures and development [114]. They found that BDNF is necessary for seizure-induced upregulation of KCC2 glycosylation but not for developmental upregulation. These findings suggest that BDNF plays a critical role in the regulation of KCC2 glycosylation during seizures. In another study by Lee et al., the effect of NMDA receptor activity on KCC2 glycosylation and GABAergic signaling was explored [100]. The researchers discovered that NMDA receptor activity downregulates KCC2 glycosylation, resulting in depolarizing GABAergic currents. These results suggest that NMDA receptor activity can modulate the balance of excitation and inhibition in the brain by altering KCC2 glycosylation. Gauvain et al. investigated the role of KCC2 glycosylation in regulating the content and lateral diffusion of AMPA receptors in dendritic spines [126]. Their findings indicated that KCC2 glycosylation is important for maintaining the correct number of AMPA receptors in spines and for regulating their lateral diffusion. Additionally, Gagnon et al. investigated the potential of chloride extrusion enhancers (CEE) as a therapeutic approach for neurological diseases [127]. The study demonstrated that CEE can enhance KCC2 glycosylation and restore inhibitory neurotransmission in animal models of epilepsy and neuropathic pain. These results suggest that CEE may be a promising therapeutic strategy for neurological disorders associated with impaired KCC2 glycosylation. Further research is needed to explore the full potential of CEE as a treatment option and to better understand its mechanism of action in enhancing KCC2 glycosylation.

#### KCC2 ubiquitination regulation

KCC2 ubiquitination dysfunction has been implicated in various conditions, including general anesthesia, epilepsy, neuropathic pain, and autism spectrum disorders. Hu et al. made a significant finding regarding the physical interaction between KCC2 and the ubiquitin ligase Fbxl4 in regulating KCC2 expression in the VPM of the thalamus. This interaction serves as a key mechanism in the recovery of consciousness from anesthesia [97] (for detailed discussions, refer to Section 3.1.3). Chen et al. identified a physical interaction between KCC2 and amyloid precursor protein (APP) [128]. Deficiency of APP leads to notable reductions in both the total and membrane levels of KCC2, resulting in a shift in E_GABA_ towards depolarization. By restoring the normal expression and function of KCC2 in *App*^*–/–*^ mice, E_GABA_, GABA_A_R α1 levels, and GABA_A_R-mediated phasic inhibition were rescued. These findings indicate that APP acts to limit tyrosine-phosphorylation and ubiquitination processes, thereby preventing the subsequent degradation of KCC2. This mechanism explains how APP influences the abundance of KCC2. In the study by Ma et al., they investigated the effects of BDNF treatment on KCC2 ubiquitination in the dorsal horn of adult mice [129]. They demonstrated that direct administration of BDNF to the spinal cord enhances the interaction between KCC2 and Casitas B-lineage lymphoma b (Cbl-b), an E3 ubiquitin ligase involved in nociceptive information processing. Knocking down Cbl-b expression resulted in decreased KCC2 ubiquitination levels and a reduction in BDNF-induced pain hypersensitivity. Additionally, they observed that following spared nerve injury, KCC2 ubiquitination significantly increased, but this effect could be reversed by inhibiting the TrkB receptor. These findings suggest that Cbl-b plays a crucial role in modulating KCC2, an important substrate related to pain, and that ubiquitin modification contributes to the impairment of KCC2 function induced by BDNF in the spinal cord. Goutierre et al. investigated the role of KCC2 in regulating neuronal excitability and hippocampal activity through its interaction with Task3 channels [130]. They discovered that KCC2 and Task3 channels interact to modulate neuronal excitability, which is crucial for proper hippocampal activity. Moreover, they found that KCC2 ubiquitination plays a role in this interaction. These findings suggest that targeting this interaction could be a potential therapeutic strategy for neurological disorders. Further research is needed to fully understand the therapeutic implications of KCC2 ubiquitination and its potential for treating various neurological conditions.

#### Sonic hedgehog (Shh) signaling

Sonic hedgehog (Shh) and its receptor complex, patched-smoothened, play crucial roles in neural stem cell proliferation and differentiation within the developing CNS. Our recent findings indicate that activated Shh signal transducer smoothened (Smo) signaling during development accelerates the transition from depolarizing to hyperpolarizing GABA, a process that relies on the functional expression of KCC2 [131]. Previous studies have reported increased expression of Shh in epileptic patients and animal models [132], as well as in the temporal neocortex in response to epileptiform discharge [133]. Collectively, these studies suggest that Shh/Smo signaling regulates chloride homeostasis and contributes to the onset of epilepsy by modulating KCC2 cell-surface expression and neuronal activity. Exploring specific antagonists of the Shh/Smo signaling pathway in future research could, therefore, present a novel strategy for the treatment of epilepsy.

#### mZnR/GPR39- SNAP23 signaling

Neuronal presynaptic mossy fibers release zinc ions (Zn^2+^) synaptically, triggering intracellular Ca^2+^ signaling through the activation of a postsynaptic metabotropic Gq-protein-coupled receptor known as mZnR/GPR39. It has been discovered that the KCC2 C-terminal domain plays a crucial role in the regulation of Zn^2+^-dependent transport through mZnR/GPR39 signaling. SNAP23, a SNARE protein associated with synaptosomes and an integral component of the membrane insertion machinery, interacts with KCC2 and enhances its activity [134]. In hippocampal neurons, mZnR/GPR39 increases the binding of SNAP23 to KCC2, thereby promoting its surface expression [134]. This process relies on the phosphorylation activation of SNAP23 by IκB kinase (IKK), as demonstrated by the prevention of mZnR/GPR39-induced upregulation of KCC2 activity through SNAP23 phosphorylation-insensitive mutants or pharmacological inhibition of IKK [134]. The study suggests that IKK could also serve as a potential drug target for modulating KCC2 activity in the treatment of epilepsy.

#### PACSIN1- KCC2 signaling

In a study exploring the KCC2 interactome, it was discovered that Protein kinase C and casein kinase substrate in neurons protein 1 (PACSIN1) serves as a novel native binding partner of KCC2, exerting a negative regulatory effect on KCC2 expression and function in hippocampal neurons [135]. Knockdown of PACSIN1 through shRNA in hippocampal neurons resulted in increased KCC2 expression and hyperpolarization of the reversal potential for Cl^-^ [135]. However, it remains unknown whether PACSIN1-mediated reductions in KCC2 are associated with any specific neurological disorders.

#### SERBP1 and IGF-1

Serpine mRNA binding protein 1 (SERBP1) and the Ago2/miR-92 complex have been found to bind to the 3ʹUTR of KCC2, potentially playing a significant role in modulating KCC2 translation in SH-SY5Y cells and primary hippocampal neurons [136]. Additionally, insulin-like growth factor (IGF-1) and the neuropeptide oxytocin (OXT) have been identified as regulators of KCC2 function. In methyl CpG binding protein 2 (MECP2) knockout mice, which exhibit KCC2 expression deficits and synaptic transmission impairments, the administration of recombinant full-length human IGF-1 (rhIGF-1) or OXT pharmacologically rescued these effects [137]. Consistent with these findings, another study demonstrated that adeno-associated viral overexpression of IGF-1 (AAV-IGF-1) in female Sprague-Dawley rats resulted in elevated levels of KCC2 and improved motor function after spinal cord injury [138]. These findings suggest the existence of potential new modulators of KCC2.

## Pharmacological modulation of KCC2

Overall, the previously discussed literature demonstrates the involvement of reduced functional activity of KCC2 in epileptic activity. This has raised the possibility of pharmacologically enhancing KCC2 as a crucial therapeutic strategy for epileptic patients. The underlying concept is that enhancing KCC2 activity prevents the accumulation of intracellular Cl^-^, thereby preventing the loss of neuronal inhibitory GABAergic signaling associated with hyperexcitability [127]. So far, four different categories of KCC2 modulators have been identified: (1) KCC2 inhibitors or antagonists, (2) KCC2 activators or agonists, (3) indirect KCC2 modulators, and (4) KCC2 genetic modulators (Table 2).

### Neuronal hyperexcitability results from KCC2 antagonism

A decrease in KCC2 activity has been recognized as one of the primary causes of hyperexcitability associated with epilepsy, given KCC2’s functional contribution to maintaining excitation-inhibition balance [139, 140]. The pathogenic role of KCC2 dysfunction has been extensively studied by employing the potent and selective KCC2 inhibitor, VU0463271 (Table 2) [104, 139–143]. VU0463271, discovered by Delphire et al., has shown enhanced selectivity for KCC2 compared to previously utilized pharmacological agents like furosemide [144]. Examining the literature on the effects of KCC2 antagonism on epileptic activity can help determine the potential of KCC2 as a therapeutic treatment for epilepsy.

Perforated-patch recording with gramicidin is suitable for studying anionic channels and their regulation of homeostatic [Cl^-^]_i,_ because this type of electrophysiological method can avoid artifactual changes in intracellular chloride concentration [145]. The application of VU0463271, both in vitro and in vivo, has been shown to result in increased neuronal excitability [140, 141]. In vitro gramicidin perforated-patch recordings of muscimol-induced currents from rat reticular thalamic (RT) neurons in the presence of VU0463271 (10 μM) resulted in a more depolarized E_GABA_ of RT neurons compared to baseline (−58 ± 3.4 mV to −42 ± 5.4 mV, t_(4)_ = 6.24) [140]. Similarly, neurons within the ventrobasal thalamus (VB) also exhibited a change in E_GABA_, with an approximate positive shift of 17 mV (−69 ± 5.6 mV to −52 ± 8.7 mV, t(4) = 7.47) [140]. These findings highlight the importance of KCC2 in maintaining GABAergic inhibition and Cl^-^ homeostasis, even when KCC2 expression is low. Immunofluorescence studies indicate low KCC2 expression in RT neurons [140]. These findings are consistent with a previous study conducted by Sivakumaran et al. in 2015 using the same electrophysiology technique but with cultured hippocampal neurons [141]. Sivakumaran et al. found that the application of VU0463271 (10 μM) resulted in a depolarizing shift of E_GABA_ from −76 ± 5 mV to −36 ± 2 mV [141]. However, the positive shift in E_GABA_ is greater in this study in comparison to Klein et al.’s. This difference may be attributed to Sivakumaran and colleagues using cultured mouse neurons instead of rat neurons. Overall, the literature demonstrates that KCC2 antagonism leads to neuronal excitability by switching GABA-mediated inhibition to GABA-mediated excitation.

**Fig. 4:**
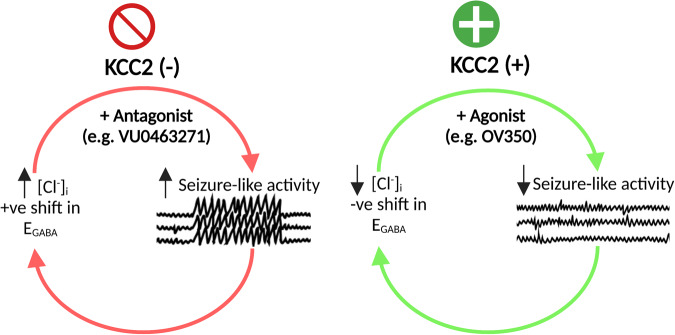
The reciprocal effect of KCC2 antagonist VU0463271 and KCC2 agonist OV350 on epileptiform activity. The KCC2 antagonist VU0463271 increases intracellular Cl^-^ concentration by reducing KCC2-dependent Cl^-^ extrusion. This elevated intracellular chloride leads to a positive, depolarizing shift in E_GABA_ in vitro. In vitro and in vivo studies have demonstrated that VU0463271 enhances seizure-like activity, including interictal and ictal events. In contrast, the KCC2 agonist OV350 reduces intracellular Cl^-^ concentration, restoring Cl^-^ to homeostatic levels and resulting in a negative shift in E_GABA_. OV350’s promotion of KCC2 activity and subsequent reduction in intracellular Cl^-^ concentration have been shown to decrease seizure-like activity, including ictal events and duration. The diagram was created using BioRender.com. KCC2 K^+^-Cl^-^−cotransporter 2, GABA γ-aminobutyric acid, E_GABA_ GABA_A_R-medicated currents, and [Cl^-^]_i_, intracellular chloride concentration.

Research has shown that KCC2 antagonism exacerbates neuronal hyperexcitability, both ex vivo and in vivo in the brain, as depicted in Fig. 4 [139]. Ex vivo electrophysiology employed to assess the impact of abolished KCC2 activity by VU0463271 revealed that VU0463271 (10 μM) increased spiking frequency of CA1 pyramidal neurons [139]. In vivo, particularly under hypoxic conditions, there was an increase in spike and sharp wave activity was observed during the interictal period (the epileptiform activity between seizures), as well as a rise in the number of ictal events (the epileptiform activity during a seizure) [139]. These findings hold high validity due to Raol and colleagues’ utilization of video and EEG recordings to observe the rats’ seizure-like activity, enabling researchers to establish correlations between seizure patterns and behavioral/motor changes. Consequently, this eliminates the possibility of the seizure-like activity being mere artifacts [146]. However, it is worth noting that the hippocampal neuron slices used in the study were obtained from male rats aged between P7-P9. Considering the developmental increase in KCC2, which is considered to be fully operational by P10–15 [11], conducting the study using older hippocampal slices might have yielded an even greater impact of VU0463271, thus resulting in more severe epileptic-like activity. This research, therefore, signifies the role of KCC2 in limiting overexcitation.

Evidence suggests that the application of VU0463271 results in continuous epileptic activity [104, 141, 143]. This has been demonstrated in acute mouse hippocampal brain slices exposed to 0-Mg^2+^ conditions, an in vitro epilepsy model [104]. VU0463271 (10 μM) induced continuous clonic-like discharges (seizure activity associated with repeated muscle movements such as jerking) and increased the duration of seizure-like events [104]. This continuous epileptiform activity is consistent with previous research using a similar methodology, while this research also suggests that VU0463271 increases the duration of ictal events [141]. Further support for VU0463271 altering epileptic activity has been demonstrated in an in vitro 4-Aminopyridine (4-AP) epileptic model, indicating KCC2 antagonism results in a change from low voltage fast-activity (ictal events) to continuous interictal spiking [143]. The continuous interictal events reflect the neuronal hyperexcitability caused by the downregulation of KCC2. However, the previous study calls into question whether VU0463271 exacerbates both ictal and interictal seizure activity. Chen et al. found that the addition of VU0463271 (10 μM) abolishes ictal discharges induced by 4-AP, with the authors proposing that KCC2 function is required for 4-AP-induced ictal events [143]. This difference in findings may be due to methodological differences. Chen and colleagues’ use of tetrode recordings may allow more accurate spike sorting than standard field recordings conducted by Kelley et al. [104, 143, 147]. This study was also the first to use tetrode recordings within the entorhinal cortex to demonstrate that abolished KCC2 function impaired ictal discharges [143]. However, Myers et al. demonstrated that antagonism of KCC2 activity reduces 4-AP-induced ictal-like activity via 60-channel multi-electrode array recordings, highlighting the complexity of KCC2’s role in seizure-like activity and epileptic pathology [142]. Future work should focus on studying ictal activity through ictal EEGs to determine whether reduced KCC2 activity can increase ictal activity. If this is the case, it would affect the therapeutic potential of targeting KCC2. Overall, the literature suggests that KCC2 activity is required for normal physiology and has a vital role in limiting overexcitation. The research performed supports the assertion that enhancing KCC2 activity may be an effective therapeutic strategy in treating individuals with epilepsy.

### Direct KCC2 agonism

The direct enhancement of KCC2 activity through the KCC2 activator CLP257 (Table 2), discovered by Gagnon et al., has been proposed as a potential therapeutic agent [127, 148]. High-throughput screening revealed that CLP257 (200 nM) selectively increases KCC2 activity. This was demonstrated by a significant 61% increase in KCC2 transport activity in *Xenopus laevis* oocytes microinjected with complementary ribonucleic acid (cRNA) encoding these CCCs, without affecting other CCC activities [127]. Moreover, CLP257 restored Cl^-^ homeostasis in neurons with reduced KCC2 activity by rescuing its cell surface expression [127]. However, due to the short terminal half-life of CLP257, its prodrug CLP290 (Table 2) is suggested to be a more effective therapeutic agent [127]. This research indicates that CLP290 could potentially reverse the high intracellular Cl^-^ concentration associated with hyperexcitability in epilepsy resulting from reduced KCC2 activity. It is important to note that this research was conducted on spinal cord slices modeling neuropathic pain, which limits its applicability in evaluating CLP257 as a potential therapeutic agent for epilepsy. Similar limitations are observed in the current literature, which primarily focuses on CLP257 as a treatment for neuropathic pain [149]. Nonetheless, some studies have explored the effects of CLP257 on epileptic activity. Evidence suggests that CLP257, by enhancing KCC2 activity, reduced the duration and frequency of ictal-like discharges in vitro [150]. However, Hamidi and Avoli raised concerns by reporting that CLP257 increased the duration of ictal discharges using the 4-AP model of epilepsy [151]. These contrasting results may be attributed to the higher concentration of CLP257 used by Hamidi and Avoli (100 μM compared to 30 μM), which could have led to non-specific effects [151]. Additionally, it has been suggested that Hamidi and Avoli mischaracterized epileptiform events, emphasizing the need for caution in interpreting their conclusions [104, 151]. However, Hamidi and Avoli’s research underscores the importance of examining the impact of high extracellular K^+^ levels generated by elevated KCC2 activity on seizure generation [151]. The high extracellular K^+^ concentration produced in the 4-AP model could directly depolarize neurons, leading to further seizures [152]. Further research is needed to determine the potential effectiveness of KCC2 activators in epileptic patients with varying degrees of reduced KCC2 activity, considering the possibility of excessively high extracellular K^+^ levels that might depolarize neurons.

Later, the mechanism of CLP257 as a KCC2 activator has been challenged by Cardarelli et al., who proposed that CLP257 may exert its effects by potentiating GABA_A_Rs instead [153]. This raises the need for a reinterpretation of the previously discussed studies. However, there have been questions regarding the replicability of Cardarelli et al.’s findings [3]. This underscores the necessity for additional studies to investigate the pharmacodynamic profile of CLP257 (and CLP290) before evaluating their potential therapeutic effects in a clinical setting. Encouragingly, preclinical toxicological studies involving the administration of CLP290 to rats have revealed no adverse side effects, indicating the possibility of evaluating the therapeutic effectiveness of CLP290 in clinical trials [127]. Recently, the mechanism of action of CLP290 to reverse or reduce epileptic activity has been described, showing that CLP290 acts by restoring the phosphorylation of Ser940, resulting in increased KCC2 membrane localization in Tat^+^ mice [154]. Recently, Zuo et al. developed a microinvasive nanodrug delivery system using reactive oxygen-responsive copolymers and neurotransmitter-conjugated CLP257 [155]. This microinvasive approach results in significant functional improvement in rats with contusive spinal cord injury. Ultimately, further research conducted on human neuronal lines is necessary to determine the safety of pharmacological activators and to translate the promising preclinical findings of CLP257 or CLP290 into a viable therapeutic agent.

Recently, Jarvi and colleagues identified a compound, Cmp1, by screening 1.3 million compounds using an established thallium (TI) influx assay [156]. Cmp1 was found to enhance KCC2 activity at an EC_50_ of 2.01 μM, with no effect on the activity of KCC3, KCC4, or NKCC1. Building upon Cmp1, they optimized the compound through medicinal chemistry, resulting in the derivative OV350 (350). OV350 exhibited an EC_50_ of 261.4 nM for KCC2 without altering its plasma membrane accumulation and phosphorylation, which are important for KCC2 function [75, 99, 157]. To observe the drug effect of OV350, Jarvi and colleagues conducted gramicidin perforated patch-clamp recordings on 18–21 DIV hippocampal neurons expressing high levels of KCC2 [71]. They used bumetanide and tetrodotoxin during the recordings to limit the effects of NKCC1 and activity-dependent changes in Cl^-^ levels, respectively [100]. Administration of 300 nM OV350 significantly decreased intracellular Cl^-^ levels, as evidenced by a decrease in E_GABA_ from −75 ± 3.1 to −85.1 ± 4.2 mV and a reduction in intracellular Cl^-^ from 7.7 ± 0.4 to 5.1 ± 0.4 mM. Conversely, the vehicle did not have a significant impact on E_GABA_, suggesting that KCC2 activation by OV350 is responsible for the observed changes in intracellular Cl^-^ levels. To evaluate the compound’s impact on KCC2 activity in a dynamic setting, cultures were exposed to OV350 for 1 h, followed by whole-cell patch-clamp recordings. Neurons were artificially loaded with 32 mM Cl^-^ via the patch pipette. The OV350-treated cells exhibited lower basal E_GABA_ values compared to the controls (−59.3 ± 2.1 vs −47.4 ± 2.3 mV), indicating a decrease in neuronal Cl^-^ levels (Fig. 4). The impact of OV350 on "seizure-like events" (SLEs) was evaluated in mouse brain slices exposed to Mg^2+^-deficient artificial cerebrospinal fluid, a method commonly used to increase neuronal excitability [158]. Field recordings within the entorhinal cortex were employed to observe the evolution of SLEs and their progression into late recurrent discharges, which resemble the development of status epilepticus [80]. OV350 did not affect the appearance of the first SLE but delayed the progression of LRDs. Additionally, the authors analyzed the interictal interval, comparing the time span between the first and second SLE. The results showed that OV350 considerably prolonged the interictal interval, and the activity levels returned to baseline after the initial SLE ended. Jarvi and colleagues also observed that subcutaneous administration of OV350 in mice resulted in significant brain penetration and CNS activity. While KCC2 activation by OV350 did not cause significant changes in mouse behavior, it provided protection against seizures induced by the GABA_A_R antagonist pentylenetetrazole and refractory seizures induced by benzodiazepines, canonical GABA_A_R-positive allosteric modulators [156]. Thus, OV350 is the first reported compound to directly bind to and activate the KCC2 cotransporter, a target implicated in neuronal excitation, including epilepsies in vivo. Nevertheless, further efforts are required to develop oral and intravenous (IV) formulations for OV350 or its analogs before proceeding to clinical trials.

### Pharmacological enhancement of KCC2 gene expression

As mentioned in Section 2.1, epileptic patients have been found to have reduced KCC2 expression [23]. Zavalin et al. conducted a study where they conditionally knocked out KCC2 in Dlx5-lineage neurons (Dlx5 KCC2 cKO) in a mouse line, targeting cortical interneurons and post-mitotic GABAergic neurons in the forebrain during embryonic development. They found that the loss of KCC2 caused an imbalance in cortical interneuron subtypes, occasional spontaneous seizures, and early death [159]. Cheung et al. established a mouse line with 2–3-fold KCC2 overexpression occurring in pyramidal neurons. They discovered that enhanced KCC2 expression significantly reduced spike rate, time in seizure, and EEG spectral power following the administration of diazepam (5 mg/kg, i.p.), thereby increasing diazepam’s efficacy in stopping EEG seizures [160]. These in vivo studies suggest that normal KCC2 function is crucial for proper brain development, and increased KCC2 activity is beneficial for treating epilepsy. Tang et al. developed neuron-based high-throughput screening assays to identify chemical compounds that can increase KCC2 gene expression from a library of 900 small-molecule chemicals [161]. Among the library, several KCC2 expression-enhancing compounds were found, including KEEC KW-2449 (an inhibitor of tyrosine kinase 3 (or FLT3) [162]) (Table 2), resveratrol (activating the sirtuin 1 (or SIRT1) signaling pathway [163]), piperine (a transient receptor potential cation channel subfamily V member 1 (or TRPV1) activator [164]), and BIO (an inhibitor of the glycogen synthase kinase 3β (or GSK3β) pathway [165]). These compounds induced a significant increase in KCC2 expression in a dose-dependent manner in cultured human neurons[161]. Further experiments using gramicidin-perforated patch recordings revealed that KW-2449 induced a significant hyperpolarizing shift in E_GABA_ in human Rett syndrome (RTT) neurons, bringing it to a level similar to that in wild-type neurons. Additionally, KW-2449 treatment significantly enhanced the chloride extrusion rate of human RTT neurons [161]. Administration of KW-2449 (2 mg/kg) or piperine (6 mg/kg) ameliorated disease-related behavioral pathologies by reducing the frequency of breathing pauses or increasing locomotion in 4-week-old male *Mecp2* mutant mice [161]. These identified compounds that enhance KCC2 expression could be potential therapeutic agents for future epileptic treatments.

### Enhanced KCC2 expression by CRISPRa

Recently, Shi et al. utilized an adeno-associated virus (AAV)-mediated CRISPR-mediated transcriptional activation (CRISPRa) system to selectively enhance the expression of KCC2 in the subiculum [166]. This approach aimed to investigate the therapeutic potential of KCC2 in various in vivo epilepsy models. Calcium fiber photometry was employed to examine the role of KCC2 in restoring impaired GABAergic inhibition. The CRISPRa system effectively increased KCC2 expression both in cell culture (300-fold increase) and in the targeted brain region in vivo (1.7-fold increase). Delivery of CRISPRa using adeno-associated viruses led to upregulation of KCC2 in the subiculum, resulting in a reduction in the severity of hippocampal seizures and enhancing the anti-seizure effects of 1 mg/kg diazepam in a hippocampal kindling model. In a model of kainic acid-induced epilepticus, KCC2 upregulation significantly improved the termination rate of diazepam-resistant epilepticus, thereby widening the therapeutic window. Notably, KCC2 upregulation mitigated valproate (300 mg/kg)-resistant spontaneous seizures in a chronic epilepsy model induced by kainic acid. Calcium fiber photometry demonstrated that CRISPRa-mediated KCC2 upregulation partially restored the impaired GABAergic inhibition in epilepsy. These findings underscore the potential of AAV-mediated delivery of CRISPRa as a promising translational approach for treating neurological disorders. Specifically, targeting KCC2 holds significant promise in managing epilepsy, particularly as an adjunctive therapy for patients with drug-resistant epilepsy. However, it is important to note that increasing KCC2 expression through this method may only have a limited effect in restoring GABA neurotransmission, restoring it to approximately one-third of its original state. This suggests that a combination treatment utilizing CRISPR interference (CRISPRi) targeting NKCC1 may lead to improved outcomes in restoring GABA neurotransmission.

## Conclusions and future directions

KCC2 dysfunction plays a crucial role in epileptogenesis and the pathogenesis of epilepsy. The existing literature clearly indicates that normal functioning and expression patterns of KCC2 are essential for maintaining intracellular Cl^-^ concentration, thereby facilitating GABAergic inhibition in the brain, which helps prevent neuronal hyperexcitability, a prerequisite for seizure generation. However, while studies on human brain slices and genetic investigations have identified reduced KCC2 activity and expression in epileptic patients, the precise mechanisms underlying this altered activity and expression remain unclear. Preclinical research has demonstrated that overactivity of WNK-SPAK/OSR1 in phosphorylating KCC2 at Thr906 and Thr1007, BDNF-TrkB signaling in downregulating KCC2 expression, and the underactivity of PKC in phosphorylating KCC2-Ser940 increase seizure activity and susceptibility. Future research can explore the correlation between WNK, SPAK, PKC, and/or BDNF expression levels in human epileptic tissue to determine if the overexpression of these trophic factors and kinases contributes to epileptogenesis through their indirect regulation of KCC2. Genetic studies can also investigate the activity of these signaling pathways to identify genetic risk factors for epilepsy.

This review has summarized the role of KCC2 in epileptogenesis and the research investigating effective therapeutic strategies, providing an overall understanding of KCC2 as a therapeutic target. Future research should focus on utilizing pharmacological inhibitors (for WNK1, SPAK, PP1, and PLCγ1) and activators (for PKC) to determine whether enhancing KCC2 activation through modulation of KCC2 Thr906, Thr1007, and Ser940 is a clinically effective anticonvulsant strategy. It is important to note that most of the research conducted on KCC2 modulation has been carried out on animal neuronal cell lines, which may have a different neuronal phenotype than human neuronal cell lines. Continued research using human tissue is necessary to determine the applicability of the various therapeutic strategies discussed. Furthermore, future research is needed to investigate the potential of KCC2 modulation in preventing epileptogenesis rather than solely reducing seizure activity and severity. Such research would unveil the full potential of targeting KCC2 in epilepsy treatment.

KCC2 demonstrates promising potential as a therapeutic target for epilepsy. Direct modulation of KCC2 activity or indirect enhancement of activity by decreasing Thr906 and Thr1007 phosphorylation, increasing Ser940 KCC2 phosphorylation, and increasing KCC2 transcription show promising anticonvulsant effects by effectively reducing seizure activity. Additionally, indirect KCC2 modulation via the inhibition of WNK-SPAK/OSR1 and BDNF-TrkB signaling holds promise as an adjunct therapeutic strategy for drug-resistant epileptic patients. Previous studies have indicated that both kinases, WNK and SPAK/OSR1, within the WNK-SPAK/OSR1 complex, directly regulate the Thr1007 site of KCC2. However, the direct kinase responsible for the Thr906 site remains to be identified in future studies, which could facilitate drug discovery for targeting the KCC2 Thr906 site. OV350 is a potential first-in-class KCC2 direct activator that exhibits significant efficacy in reducing neuronal excitation, included in the treatment of epilepsies, as demonstrated in in vivo studies. Nevertheless, additional efforts are needed to develop oral and IV formulations of OV350 or its analogs before they can proceed to clinical trials. The application of AAV-mediated delivery of CRISPRa has been employed to partially upregulate KCC2 and restore impaired GABA-mediated inhibition in epilepsy. This approach holds significant promise for managing epilepsy, particularly as an adjunctive therapy for patients with drug-resistant epilepsy. However, the therapeutic effect is somewhat limited in terms of restoring GABA neurotransmission. Hence, a combination treatment involving CRISPRi targeting NKCC1 may potentially yield enhanced outcomes in restoring GABA neurotransmission. Alternatively, the dual regulator ZT-1a, which directly targets the shared upstream kinase of both KCC2 and NKCC1, may potentially achieve improved drug effectiveness for epilepsy treatment.
